# A machine learning classifier to identify and prioritise genes associated with murine cardiac development

**DOI:** 10.1371/journal.pgen.1011489

**Published:** 2026-02-10

**Authors:** Mitra Kabir, Verity Hartill, Gist H. Farr III, Wasay Mohiuddin Shaikh Qureshi, Stephanie L. Baross, Andrew J. Doig, David Talavera, Michael R. Waterfield, Bernard D. Keavney, Lisa Maves, Colin A. Johnson, Kathryn E. Hentges

**Affiliations:** 1 Division of Evolution, Infection and Genomics, Faculty of Biology, Medicine and Health, Manchester Academic Health Science Centre, British Heart Foundation Centre of Research Excellence, The University of Manchester, Manchester, United Kingdom; 2 Leeds Institute of Medical Research, University of Leeds, St James University Hospital, Leeds, United Kingdom; 3 Yorkshire Regional Genetics Service, Leeds Teaching Hospitals NHS Trust, Chapel Allerton Hospital, Leeds, United Kingdom; 4 Centre for Developmental Biology and Regenerative Medicine, Seattle Children’s Research Institute, Seattle, Washington, United States of America; 5 Division of Cardiovascular Sciences, School of Medical Sciences, Faculty of Biology, Medicine, and Health, The University of Manchester, Manchester, United Kingdom; 6 Division of Molecular and Cellular Function, School of Biological Sciences, Faculty of Biology, Medicine and Health, University of Manchester, Manchester, United Kingdom; 7 Department of Pediatrics, University of California San Francisco, San Francisco, California, United States of America; 8 Manchester Heart Institute, Manchester University NHS Foundation Trust, Manchester Academic Health Science Centre, British Heart Foundation Centre of Research Excellence, Manchester, United Kingdom; 9 Department of Pediatrics, University of Washington School of Medicine, Seattle, Washington, United States of America; University of Lausanne, SWITZERLAND

## Abstract

Congenital heart disease (CHD) is a major cause of infant mortality and presents life-long challenges to individuals living with these conditions. Genetic causes are known for only a minority of types of CHD. Discovering further genetic causes is limited by challenges in prioritising candidate genes. We examined a wide range of features of mouse genes, including sequence characteristics, protein localisation and interaction data, developmental expression data and gene ontology annotations. Many features differ between genes needed for cardiac development and non-cardiac genes, suggesting that these two gene types can be distinguished by their attributes. We therefore developed a supervised machine learning (ML) method to identify *Mus musculus* genes with a high probability of being involved in cardiac development. These genes, when mutated, are candidates for causing human CHD. Our classifier showed a cross-validation accuracy of 81% in detecting cardiac and non-cardiac genes. From our classifier we generated predictions of the cardiac development association status for all protein-coding genes in the mouse genome. We also cross-referenced our predictions with datasets of known human CHD genes, determining which are orthologues of predicted mouse cardiac genes. Our predicted cardiac genes have a high overlap with human CHD genes. Thus, our predictions could inform the prioritisation of genes when evaluating CHD patient sequence data for genetic diagnosis. Knowledge of cardiac developmental genes may speed up reaching a genetic diagnosis for patients born with CHD.

## Author summary

Congenital heart disease arises during pregnancy when the heart has formed incorrectly. These malformations affect ~1% of newborns. Yet, despite their frequency, the underlying causes are still not known in many cases. Genetic factors are a significant cause of CHD, and increasingly DNA sequencing is used to attempt to determine if an individual has a genetic change causative of their condition. However, analysis of patient sequence data often reveals changes that are difficult to interpret due to a lack of knowledge of the function of the gene harbouring a sequence change. We aimed to facilitate the process of sequence evaluation by predicting which genes of unknown function are likely involved in heart formation. Our predictions agree well with novel experimental evidence about genes needed for heart development, as we found that when mutated, a high proportion of the predicted cardiac genes do indeed cause heart defects. This result suggests that our predictions may be informative for expanding our understanding of the genetic basis of congenital heart disease.

## Introduction

Congenital heart disease (CHD), resulting from errors in the formation of the heart during gestation, is the most common structural malformation present at birth, affecting approximately 1.35 million infants annually worldwide [1], and is the primary contributor to infant mortality arising from birth defects [2]. CHD includes a wide variety of cardiac and great vessel malformations with varying severity in phenotype [3]. Advances in detection and surgical interventions have led to increased survival rates among children with CHD; however, patients seem to develop a wide range of co-morbidities. For example, psychological illness persists as a significant co-morbidity in adolescents and adults affected by the condition [4]. Children with CHD have a higher rate of neurodevelopmental disorders than their peers without CHD, and at 22 months of age score significantly lower on measures of cognitive, motor and language development than the standardised population mean [5]. Additionally, children and young adults with CHD face an elevated risk of developing cancer [6]. Hence, CHD not only impairs cardiovascular function but also causes co-morbidities that profoundly affect quality of life and life expectancy.

Environmental factors may contribute to abnormal cardiac development [7]. However, due to the complexity in genetic and cellular interactions needed for the heart to form properly, perhaps it is not surprising that many cases of CHD result from pathological genetic variation. Despite much research into the underlying genetic associations with CHD, a specific genetic diagnosis is still lacking in up to 80% of sporadic CHD cases and up to 70% of familial CHD cases [8,9]. Approximately two-thirds of CHD cases occur due to complex genetic mechanisms that involve interactions between multiple genes [10], adding challenges to the process of confirming pathogenicity. Non-coding RNAs also play a role in the aetiology of CHD [11,12]. Moreover, CHD can arise from *de novo* mutations, with the absence of familial inheritance [13]. Previous studies have estimated that several hundred genes may contribute to CHD susceptibility [14]. Genome sequencing of patients with CHD has identified genetic variants in genes not previously known to play a part in cardiac development [14–16]. However, a standardised system to evaluate the significance of these variants is lacking, despite the urgent need for such knowledge to facilitate genetic diagnosis and inform genetic counselling.

Advancements in discovering further genetic causes for CHDs can be limited by the challenges in prioritising candidate genes from CHD patient sequence analyses. Comparison to mutant mouse models can aid in prioritisation, although data from mouse knockout experiments are limited. As of April 2024, the International Mouse Phenotyping Consortium (IMPC) [17] completed phenotyping for knockouts of only 8707 of the approximately 22,500 protein-coding mouse genes, leaving many genes uncharacterised. Consequently, current sequence variant prioritisation methods often fail due to a lack of data, lack of knowledge over which factors are most informative for determining pathogenicity, and challenges in manually integrating information from disparate data sources [18,19]. A new method to prioritising candidate genes is imperative, and machine learning (ML), a subset of artificial intelligence (AI), holds the potential to address this challenge.

Machine learning uses algorithms to enable computers to find patterns in data characteristic of a group and to make predictions based on those patterns. Since such approaches have not yet been applied to CHD gene prediction in mammalian species, we developed a supervised machine learning classifier to identify key characteristics of genes required for cardiac development in mice. These genes, when mutated, could serve as promising candidate disease genes for CHDs in humans. Utilising a training dataset comprising mouse genes known to be either indispensable for cardiac development or not required, we trained a Random Forest classifier to distinguish between these two classes. The efficacy of our method was validated by classifying genes independent of the training dataset. We then predicted the cardiac development association status of all protein-coding genes in the mouse genome. Large-scale bioinformatic analyses revealed functions linking the predicted cardiac genes to cardiac development. RNA-Seq data showed similar expression patterns between our predicted and known cardiac genes. Many of our predicted genes are orthologues of newly published human CHD genes, affirming that our classifier predictions do indeed include human CHD gene candidates. The knowledge of cardiac development genes may assist gene prioritisation from sequence analysis, potentially accelerating the diagnosis of CHDs.

## Results

### Datasets

The Mouse Genome Informatics (MGI) database [20] serves as a comprehensive repository for published gene data on mouse knockout phenotypes. Initially, we compiled two datasets from MGI: one containing 1415 mouse genes known to be involved in cardiac development (cardiac genes), and another containing 6808 genes known not to be involved in cardiac development (non-cardiac genes). These classifications were based on phenotype annotations derived from null alleles of targeted single-gene knockouts. Any gene whose null mutation resulted in cardiac abnormalities was included in our cardiac dataset, regardless of whether additional phenotypes were present. Accordingly, our cardiac training dataset comprised all genes reported to affect cardiac development and was not restricted to genes solely involved in cardiac development and no other systems. By relying on null phenotype data to compile our training datasets, inclusion was based on functional criteria rather than gene expression patterns. To ensure precise gene classification, genes ambiguously labelled in MGI as both cardiac and non-cardiac were manually reviewed against published literature to verify their roles in cardiac development. To focus on features specific to protein function, we limited our analysis to protein-coding genes. As a result, after excluding 173 cardiac and 235 non-cardiac genes, our final datasets comprised 1242 cardiac genes and 6573 non-cardiac genes (S1 and S2 Tables).

### Cardiac genes are different in features compared to non-cardiac genes

We gathered a wide range of features of mouse protein-coding genes, encompassing gene and protein sequence-based attributes, gene expression, gene ontology (GO) annotations, and protein-protein interaction (PPI) details. In total, we analysed 127 features (detailed in the S18 Table) to compare cardiac and non-cardiac genes and identify properties associated with cardiac development. This analysis revealed significant differences in the values of several features between the cardiac and non-cardiac datasets (Table 1).

**Table 1:** List of statistically significant features between cardiac and non-cardiac genes. The median value of each feature is reported. *P*-values were determined from the Mann-Whitney U test. Statistically significant results were evaluated based on the Bonferroni corrected *P*-value.

Features	Cardiac	Non-cardiac	*P*-value
Gene length (bp)	29,727	23,733	2.6 × 10^-7^
Exon length (bp)	3281.50	2921	4.4 × 10^-11^
Intron length (bp)	26,570	20,591	9.8 × 10^-7^
Transcript count	5	4	3.0 × 10^-3^
Exon count	10	9	2.3 × 10^-4^
Protein length (aa)	499.50	462	1.7 × 10^-8^
Molecular weight (Da)	56035.56	51523.66	2.2 × 10^-8^
Aliphatic (%)	27.29	27.81	6.8 × 10^-7^
Leu (%)	9.54	9.97	2.1 × 10^-8^
Asn (%)	3.65	3.46	4.0 × 10^-6^
Pro (%)	5.85	5.62	4.4 × 10^-4^
Tyr (%)	2.87	2.75	3.1 × 10^-3^
Gene expression in heart tissues of eight-week-old mice (FPKM)	7.29	2.43	1.9 × 10^-41^
Gene expression in fibroblast tissues of eight-week-old mice (FPKM)	8.06	2.91	9.9 × 10^-26^
Gene expression in Theiler stage 10 epiblast tissues (FPKM)	2.78	1.72	1.7 × 10^-5^
Gene expression in zygote tissues (FPKM)	1.51	1.02	5.9 × 10^-5^
Gene expression in thymus tissues of eight-week-old mice (FPKM)	7.12	5.49	1.9 × 10^-5^
Gene expression in stem cell tissues (FPKM)	5.98	3.42	1.8 × 10^-11^

**Fig 1:**
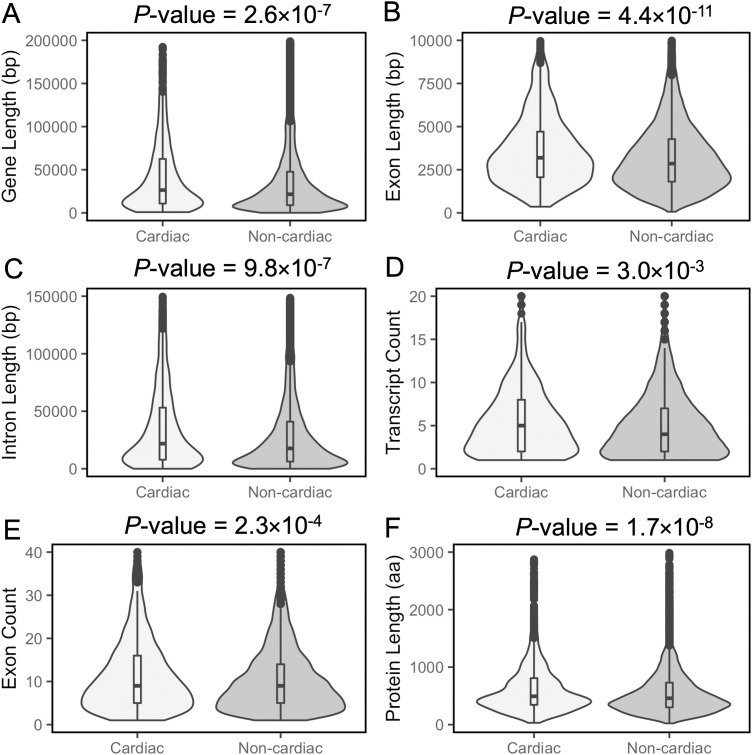
Violin plots showing the distributions of total gene length, exon length, intron length, transcript count, exon count and protein length in cardiac and non-cardiac datasets. Horizontal lines within boxplots represent median values. *P*-values were determined using the Mann-Whitney U test and adjusted for multiple comparisons with the Bonferroni correction.

We observed that cardiac genes exhibit distinct characteristics compared to non-cardiac genes, as depicted in Table 1 and Fig 1. Cardiac genes are more likely to be long, have multiple transcripts, a greater number of exons, and possess longer exons and introns. These features likely reflect the involvement of cardiac genes in complex regulatory networks that govern heart development. Longer gene sequences may facilitate extensive alternative splicing and harbour regulatory elements within intronic regions, enabling precise spatiotemporal control of gene expression during heart development. Additionally, it has been noted that developmental genes are more likely to contain enhancers spanning dozens of base pairs within their coding exons, a feature that may underlie the longer average exon length in the genes we identified as required for cardiac development [21]. It has been suggested that intron length serves as an additional means of transcription control, whereby delays in gene transcription due to longer introns is particularly important to controlling the expression dynamics of developmental genes [22]. Genes expressed after zygotic genome activation in bovine embryos tend to be longer and contain longer introns than genes expressed just after fertilisation [23], an attribute that is likely shared with cardiac development genes. Genes needed for cardiac muscle development have been specifically found to have co-evolution of their intron length [24], which may then reinforce the prevalence of longer introns in this gene category.

A higher proportion of cardiac genes are expressed during critical stages of mouse development, including blastocyst (44.7% vs. 39.5%, Chi-squared *P-*value = 8.2 × 10^-3^), gastrula (42.4% vs. 37.4%, Chi-squared *P-*value = 8.2 × 10^-3^), organogenesis (72.1% vs. 61.3%, Chi-squared *P-*value = 1.1 × 10^-5^), and neonate (80.8% vs. 73.1%, Chi-squared *P-*value = 3.5 × 10^-3^) stages, compared to non-cardiac genes. Additionally, cardiac genes demonstrate higher expression levels in various tissues, including heart tissues of eight-week-old mice, fibroblast tissues of eight-week-old mice, Theiler stage 10 epiblast, zygote, thymus tissues of eight-week-old mice, and stem cell tissues (Table 1).

**Fig 2:**
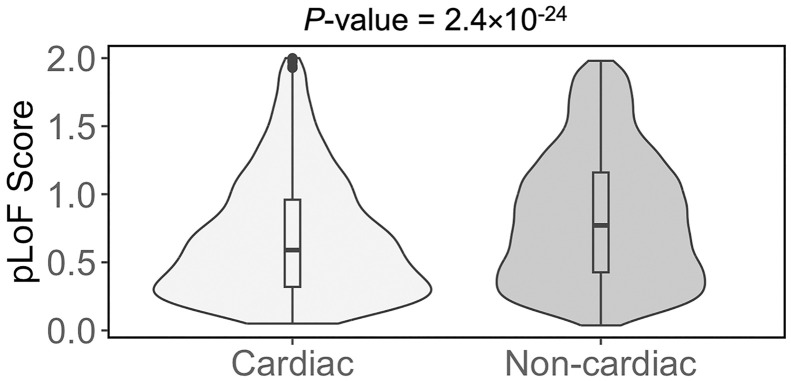
Violin plots showing the distributions of pLoF score in cardiac and non-cardiac datasets. Horizontal lines within boxplots represent median values. *P*-values were determined using the Mann-Whitney U test and evaluated for significance with Bonferroni correction.

Moreover, cardiac genes are characterised by having low probability of loss-of-function (pLoF) scores in gnomAD, as illustrated in Fig 2. The gene pLoF score is a metric used to assess the likelihood of observing variants resulting in loss-of-function of a particular gene, based on the data collected in gnomAD v2.1.1. Therefore, a low pLoF score means a gene is less likely to have loss-of-function variants present in sequence data compiled in gnomAD.

Analyses of mouse protein sequence data revealed that cardiac genes are more likely to encode proteins with longer lengths (Fig 1f) and higher molecular weights (Table 1). Longer proteins often contain additional functional domains, enabling a broader range of cellular functions and facilitating more protein-protein interactions [25].

Cardiac proteins exhibit higher proportions of Asparagine (Asn), Proline (Pro), and Tyrosine (Tyr) residues, while non-cardiac proteins display higher proportions of aliphatic and Leucine (Leu) residues (Table 1). Moreover, our analysis revealed that cardiac proteins exhibit enrichment in specific functional annotations compared to non-cardiac proteins. Cardiac proteins are more likely to function as oxidoreductases (4.8% vs. 3.2%, Chi-squared *P-*value = 7.9 × 10^-3^), transcription factors (19.1% vs. 11.9%, Chi-squared *P-*value = 1.9 × 10^-10^), to be phosphorylated proteins (58.2% vs. 46.3%, Chi-squared *P-*value = 3.2 × 10^-8^) and acetylated proteins (20.6% vs. 15.7%, Chi-squared *P-*value = 1.1 × 10^-4^), and have a higher frequency of signal peptide motifs (28.1% vs. 22.4%, Chi-squared *P-*value = 1.3 × 10^-4^). Furthermore, subcellular localisation analysis utilising UniProt annotation revealed that a greater percentage of cardiac proteins are localised within the nucleus (33.1%. vs 27.1%, Chi-squared *P-*value = 2.6 × 10^-4^) and extracellular region (16.4% vs 10.9%, Chi-squared *P-*value = 2.5 × 10^-7^) compared to non-cardiac proteins, which are mostly localised to the cytoplasm and membrane. The enrichment of nuclear and extracellular proteins among cardiac developmental genes likely reflects their roles in transcriptional regulation and intercellular communication, respectively—two processes critical to cardiac morphogenesis. It has previously been noted that transcription factors, which have a nuclear localisation, are enriched in datasets of essential proteins [26], a category that would include many proteins needed for cardiac development.

**Fig 3:**
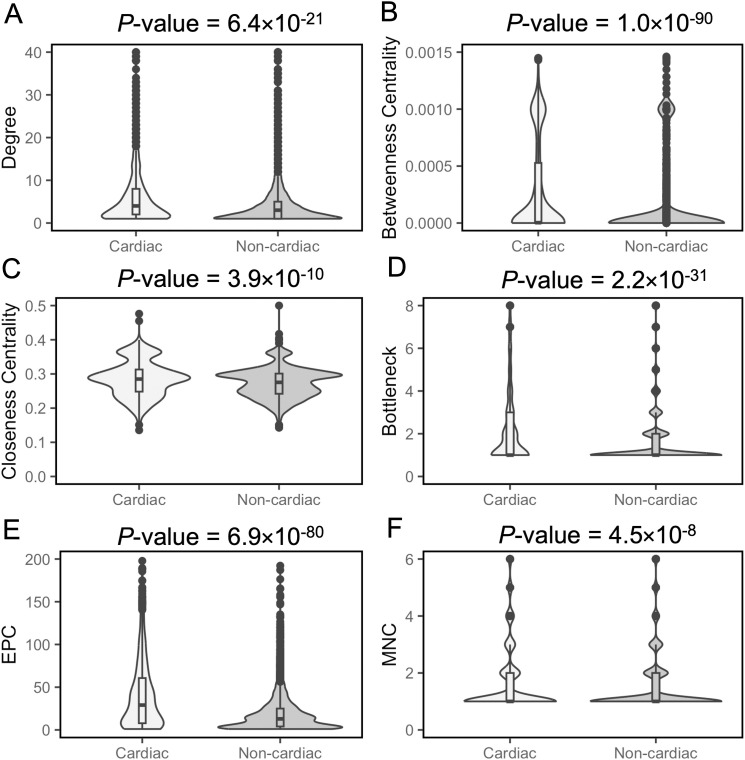
Violin plots showing the distributions of PPI network features of cardiac and non-cardiac proteins. Degree (A), Betweenness centrality (B), Closeness centrality (C), Bottleneck (D), Edge Percolation Component (E) and Maximum Neighbourhood Component (F) of cardiac and non-cardiac proteins in the PPI networks. Horizontal lines within boxplots represent median values. *P*-values were determined using the Mann-Whitney U test and evaluated for significance with Bonferroni correction.

There is an emerging view that additional genes associated with biological functions and phenotypes can be identified due to “guilt by association” with genes already known to participate in those processes [27–32]. Most approaches investigating gene associations have used protein interaction data [28–32], although others have used similarity of functional annotation [27]. In our study, we analysed the PPI data for mouse proteins from the I2D database [33] to determine whether the cardiac PPI network exhibits distinct network properties compared with the non-cardiac network. We focused solely on known mouse PPIs to ensure the inclusion of high-quality interactions. For each cardiac and non-cardiac protein, we computed 13 network properties to assess their significance within their respective PPI networks. Cardiac proteins demonstrated a higher number of interactions (higher degrees) compared to non-cardiac proteins within their interaction network (Fig 3A). Moreover, the betweenness centrality, serving as an indicator of a protein’s centrality within the PPI network, was significantly higher for cardiac proteins than non-cardiac proteins (Fig 3B). Additionally, we observed significantly higher closeness centrality values for cardiac proteins compared to non-cardiac proteins (Fig 3C).

To identify protein nodes with large number of interactions (hubs) in the PPI network, we utilised the Hub object Analyzer (Hubba) [34]. This tool allowed us to explore four additional network properties: BottleNeck (BN), Edge Percolation Component (EPC), Maximum Neighbourhood Component (MNC), and Density of Maximum Neighbourhood Component (DMNC). These properties define probable hubs, or highly connected proteins, in the interaction network. Our investigation revealed that cardiac proteins tend to have high BN, EPC and MNC values compared to non-cardiac proteins, as illustrated in Fig 3.

We analysed the Gene Ontology (GO) terms [35] associated with genes in our datasets using the web-based tool DAVID [36], as GO is the most widely used approach for annotating gene functions. Our analysis revealed notable differences in the GO term annotations for biological process and cellular component classes between cardiac and non-cardiac gene groups. For biological processes, GO data analysis demonstrated that cardiac genes are primarily involved in processes related to “heart development”, “heart morphogenesis”, “angiogenesis”, “vasculogenesis”, “heart looping” and “in utero embryonic development” (S3 Table). In contrast, non-cardiac genes are predominantly associated with processes like “immune system response”, “ion transport”, “cell adhesion”, “inflammatory response”, “cell adhesion” and “brain development” (S4 Table). This result also validates the selection of our cardiac gene dataset. For cellular component, terms most frequently associated with cardiac genes include “cell surface”, “extracellular region”, “plasma membrane” and “nucleus”. On the other hand, the non-cardiac dataset exhibited enrichment for terms such as “membrane”, “glutamatergic synapse”, “cytoplasm”, “synapse”, “cell junction”, “neuron projection”, and “cytosol”. Detailed lists of the 10 most enriched GO terms for cellular components are provided in S5 and S6 Tables.

### Processing datasets for machine learning

**Fig 4:**
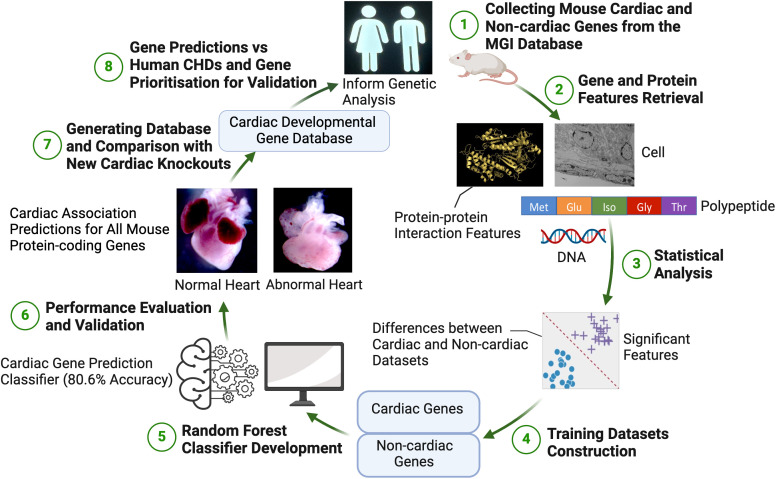
The workflow for predicting mouse cardiac developmental genes from gene and protein sequence features using a Random Forest classification model. First, features of mouse genes are collected from publicly available databases. Subsequently, statistical analyses and feature selection were performed to pinpoint the most informative features distinguishing between known cardiac and non-cardiac genes. A Random Forest classifier was constructed to predict cardiac and non-cardiac genes. Finally, this classifier was deployed to predict cardiac development association status for all protein coding genes within the mouse genome which were not used in developing the classifier. A database of prediction results has been developed and is available to the public. This figure was created in BioRender [39].

We identified distinctive features that could effectively discriminate between cardiac and non-cardiac genes. We therefore sought to develop a machine learning classifier that could categorize a mouse gene as either cardiac or non-cardiac based on these features (Fig 4). Using 127 features as input, we generated training datasets for classification. The quality of the training dataset is crucial for the success of machine learning classifiers. Initially, our dataset included 1242 cardiac genes and 6573 non-cardiac mouse genes, resulting in a significantly imbalanced class frequency ratio of 1:5.3. To mitigate the impact of class imbalance in the training dataset, we created balanced training datasets. These included all 1242 cardiac genes and an equal number of randomly selected non-cardiac genes (1242 out of 6573 total non-cardiac genes). This approach ensured our classifier was effectively trained without bias towards the majority class [37,38].

We also constructed test datasets comprising mouse genes not included in the training datasets to assess the accuracy of our machine learning classifier. Two test datasets were compiled for the validation of our predictions. Test dataset 1 (S7 Table) consists of 5331 mouse knockout genes selected randomly from our non-cardiac dataset, which were not included in our initial training set selection. Test dataset 2 (S8 Table) contains 966 mouse reporter line mutants. We restricted our training datasets to genes with mouse targeted deletion alleles only, so genes with reporter mutant lines were excluded from the compilation of our training datasets. However, phenotypic data are available in MGI for these reporter lines, and therefore they can be used as a second test set of genes with experimentally known cardiac or non-cardiac development status.

### Performance of the random forest classifier

To develop our classifier, we employed the Random Forest (RF) [40] method implemented using open source machine learning software in WEKA [41], to construct a machine learning classifier aimed at identifying genes likely to be involved in cardiac development. RF, an ensemble classifier composed of multiple decision tree models, has been demonstrated to exhibit high accuracy across various studies [42–46]. Our initial RF classifier (RF-1) was trained on all features of the training dataset using a 10-fold cross-validation approach. This methodology was employed to enhance the robustness of our classifier and mitigate potential overfitting issues. The cross-validation accuracy of RF-1 classifier was 79.8% (1982/2484), with 951 true-positives (TPs), 291 false-negatives (FNs), 1031 true-negatives (TNs), and 211 false-positives (FPs). The performance of this classifier was also assessed using various performance measures, the detailed values of which are presented in Table 2 for each class.

**Table 2:** 10-fold cross validation performance of the Random Forest classifiers, trained and evaluated on the training dataset. Data from before and after feature selection are presented. Here, TP = True Positive; FP = False Positive; ROC = Receiver Operating Curve; PRC = Precision-Recall Curve.

Classifiers	Accuracy (%)	Gene Class	TP Rate	FP Rate	Precision	F-Measure	ROC area (AUC)	PRC area
RF-1	79.8	Cardiac	0.766	0.170	0.818	0.791	0.870	0.866
		Non-cardiac	0.830	0.234	0.780	0.804	0.870	0.867
		Weighted average	0.798	0.202	0.799	0.798	0.870	0.866
RF-2	80.6	Cardiac	0.791	0.180	0.815	0.803	0.882	0.881
		Non-cardiac	0.820	0.209	0.797	0.809	0.882	0.879
		Weighted average	0.806	0.194	0.806	0.806	0.882	0.880

FNs, 1019 TNs, and 223 FPs. Performance metrics presented in Table 2 confirm that feature selection significantly enhanced RF classifier’s performance.

Using all features in a training dataset is not always optimal for classifier performance. Feature selection can reduce overfitting, enhance classification accuracy, and speed up training. We employed the Information Gain feature selection method in WEKA to identify the most relevant mouse gene features for classification, ultimately selecting 73 features from the original set of 127 (Tables 3 and S9). Most of these selected features exhibited statistically significant differences in values between cardiac and non-cardiac genes, confirming their discriminative potential in our study. We developed another RF classifier (RF-2) based on the 73 selected features, employing 10-fold cross-validation. The accuracy of this classifier improved to 80.6%, with 983 TPs, 259 FNs, 1019 TNs, and 223 FPs. Performance metrics presented in Table 2 confirm that feature selection significantly enhanced the RF classifier's performance.

**Table 3:** Top 10 features selected from the training dataset using the Information Gain feature selection method. Features are sorted in descending order with respect to the corresponding information gain value, with the most informative feature listed first. Many of the informative features are associated with protein interaction networks.

Information Gain	Feature	Feature Type	Observation for Cardiac Genes
0.09884	Eigenvector	PPI Network	Low
0.09187	Betweenness Centrality	PPI Network	High
0.08637	Edge Percolation Component	PPI Network	High
0.0851	Average Shortest Path Length	PPI Network	Low
0.08106	Closeness Centrality	PPI Network	High
0.04575	Degree	PPI Network	High
0.04199	Bottleneck	PPI Network	High
0.03697	Clustering Coefficient	PPI Network	High
0.03679	GO:0007507 Heart Development	Biological Process	High proportion
0.02889	Expression in 8 week-old Mouse Embryonic Heart	Developmental Expression Pattern	Expressed in high proportion
0.0208	pLoF	Gene Essentiality	Low

To assess potential overfitting of the Random Forest classifier due to the training dataset, we generated four additional balanced training datasets, each containing different subsets of non-cardiac genes. We then trained a separate Random Forest classifier on each of these datasets. The performance evaluations, detailed in S10 Table, revealed a mean accuracy of 80.1% with a standard deviation of 0.6. These results confirm that the Random Forest classifier is not biased by the choice of the training dataset.

We further sought to validate the superiority of the RF model over other models in predicting cardiac developmental genes in our datasets. To do so, we developed three additional machine learning classifiers: the J48 decision tree [47], support vector machine (SVM) [48], and gradient boosted tree (GBT) [49] classifiers. All models were trained and evaluated using the same set of selected features from the training dataset, employing a 10-fold cross-validation method to ensure fair and consistent comparisons of their classification performance. The performance of each classifier was evaluated using standard metrics such as accuracy, TPR, FPR, and AUC, with the results summarised in S11 Table. Among the tested models, the RF classifier demonstrated the best overall performance across these metrics. Therefore, we chose Random Forest as the classification method for predicting cardiac developmental genes, as it most effectively met the performance requirements for this study.

The performance of our classifier was then evaluated using two test datasets. Test dataset 1 contained all non-cardiac genes with known experimental data that had not been included in training the RF classifier; 86% of the genes (4603 out of a total of 5331 genes) were correctly identified as non-cardiac in this dataset. Moreover, our classifier showed a very strong performance on Test dataset 2, correctly identifying the cardiac development status for 89% of the genes (865 out of 966 genes total) with phenotypic data from mouse Cre reporter line experiments.

### Cardiac development associated gene prediction

We applied our RF classifier to the full list of 12,375 mouse protein-coding genes (retrieved from the MouseMine [50] database) with unknown cardiac-association status (S12 Table) and generated cardiac development status predictions for all these genes. We identified 4472 (36% of the prediction dataset) mouse genes likely to play a role in cardiac development. A total of 7901 (64% of the prediction dataset) genes are predicted not to be associated with cardiac development. We ranked these genes by the confidence level of their prediction (S13 Table). The top 15 predicted cardiac genes are listed in Table 4. The expression of the top 15 predicted cardiac genes was confirmed by RT-PCR analysis using RNA from E10.5 mouse whole embryos, E15.5 mouse embryonic hearts, and adult mouse hearts (S1 Fig). Consistent with their cardiac development prediction status, all the top 15 predicted genes exhibited expression in these tissues. Among these 15 genes, *Sp3* [51] and *Qki* [52,53] have been identified as crucial for cardiac development in mice, supporting the validity of our prediction. Autosomal dominant Robinow syndrome in humans, caused by pathogenic variants in *DVL1*, frequently includes ventricular septal defects [54], indicating *Dvl1* plays a role in cardiac development. *Dvl1* and *Dvl2* also play a critical role in outflow tract (OFT) lengthening during cardiac looping [55].

**Table 4:** Top 15 mouse cardiac developmental genes predicted by our Random Forest classifier.

Gene Symbol	Encoded protein and function	Predicted Class	Confidence
*Epn2*	Epsin-2; involved in clathrin-coated endocytosis	Cardiac	0.995
*Gne*	Glucosamine (UDP-N-acetyl)-2-epimerase; initiates the biosynthesis of N-acetylneuraminic acid (NeuAc), a key precursor for sialic acids	Cardiac	0.995
*Sp3*	Transcription factor Sp3; act as an activator or repressor depending on isoform or modifications	Cardiac	0.99
*Cdk2*	Cyclin-dependent kinase 2; involved in the control of the cell cycle	Cardiac	0.98
*Bmi1*	Polycomb complex protein BMI-1; required for maintaining the transcriptionally repressive state of many genes	Cardiac	0.98
*Ccne1*	G1/S-specific cyclin-E1; Essential for the control of the cell cycle at the G1/S (start) transition	Cardiac	0.975
*Dvl1*	Segment polarity protein dishevelled homolog DVL-1; participates in Wnt signaling	Cardiac	0.975
*Qki*	KH domain-containing RNA-binding protein QKI; regulates RNA metabolic processes	Cardiac	0.975
*Dpysl2*	Dihydropyrimidinase-related protein 2; plays a role in neuronal development and polarity	Cardiac	0.97
*Cenpc1*	Centromere protein C; plays a central role in assembly of kinetochore proteins	Cardiac	0.97
*Gna12*	Guanine nucleotide-binding protein subunit alpha-12; involved as modulator or transducer in various transmembrane signaling systems	Cardiac	0.97
*Dnmt1*	DNA (cytosine-5)-methyltransferase 1; essential for epigenetic inheritance	Cardiac	0.965
*Atf7ip*	Activating transcription factor 7-interacting protein 1; modulates transcription regulation and chromatin formation	Cardiac	0.965
*Vps26a*	Vacuolar protein sorting-associated protein 26A; acts as a component of the retromer cargo-selective complex (CSC)	Cardiac	0.965
*Enah*	ENAH actin regulator; involved in cytoskeleton remodeling and cell polarity	Cardiac	0.965

**Fig 5:**
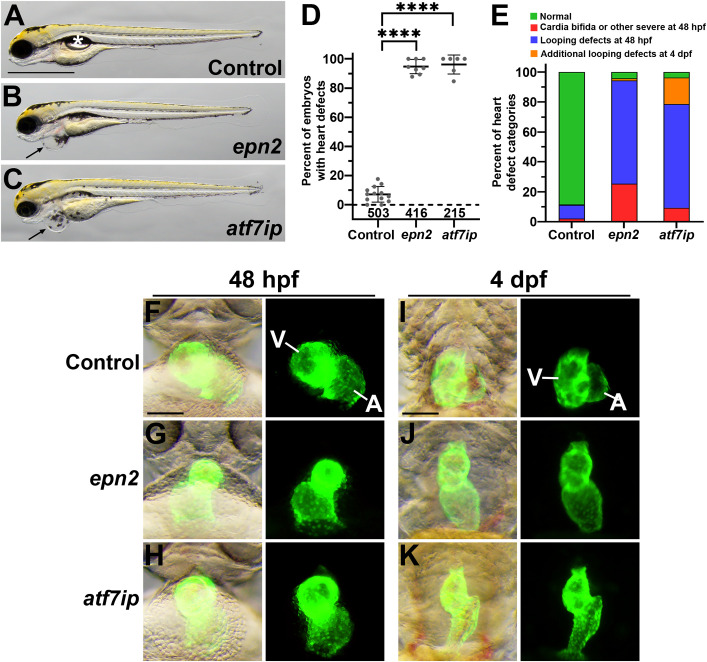
Zebrafish G0 CRISPR targeting of predicted cardiac genes causes embryonic heart defects. (A–C) Lateral views of zebrafish larvae at 4 dpf following injection at the one-cell stage with the indicated CRISPR guide RNA cocktails. The asterisk in (A) indicates the swim bladder, the formation of which is indicative of normal, healthy development. Arrows in (B) and (C) indicate pericardial edema. Scale bar = 1 mm. (D) Frequency of heart defects observed in 4 dpf larvae following CRISPR targeting. Each dot represents an independent experimental replicate batch of embryos, and bars represent the mean ± SEM. Control: N = 13 replicates; *epn2*: N = 8 replicates; *atf7ip*: N = 6 replicates. Each replicate batch comprised 9–73 embryos (mean ± SD: 42 ± 17). The total number of embryos scored is shown above the x-axis; these totals also apply to the corresponding columns in panel (E). ****P < 0.0001. (E) Frequencies of different heart defect categories observed in G0 CRISPR-targeted embryos. Cardia bifida (together with other severe defects, primarily failure of ventricle formation) and heart looping defects were scored at 2 dpf. Hearts were rescored at 4 dpf to identify defects arising between 2 dpf and 4 dpf. (F–K) Ventral views of hearts expressing GFP from a *myl7*:EGFP transgene in embryos at 48 hpf (F–H) and 4 dpf (I–K), with pigment formation inhibited; anterior is shown at the top. Representative heart phenotypes are shown, with overlaid brightfield and GFP images on the left of each image pair and the GFP channel alone on the right. Scale bar = 100 µm. V, ventricle; A, atrium.

To evaluate whether our RF classifier had identified new genes required for cardiac development, we used CRISPR/Cas9 to target the zebrafish orthologs of two of the genes from our list, *epn2* and *atf7ip*. This method uses a cocktail of four single-guide RNAs per targeted gene and complexes the guides with Cas9 protein prior to injection in 1-cell embryos to achieve a high efficiency of gene disruption, recapitulating genetic null mutations in many cases [53]. To facilitate the observation of cardiac phenotypes, we injected embryos from a line harboring the pan-myocardial transgene *myl7*:EGFP. Clutches of injected embryos were sorted for GFP expression at 24 hours post-fertilization (hpf), and heart phenotypes were scored at 48 hpf and 4 days post-fertilization (dpf). Injection of control CRISPR guides resulted in approximately 90% phenotypically normal larvae at 4 dpf (Fig 5A, 5D, and 5E). In contrast, G0 CRISPR targeting of either *epn2* or *atf7ip* consistently yielded larvae with a pronounced swelling around the heart, termed pericardial edema, which can be caused by reduced cardiac function (Fig 5B and 5C). By 4dpf, more than 90% of the *epn2* and *atf7ip* targeted embryos showed structural cardiac defects (Fig 5D and 5E). During normal zebrafish heart development, beginning at about 26 hpf, the linear heart tube bends and twists, while also forming a constriction in its middle, rearranging into an S-shaped structure at the midline with two distinct ballooned chambers arranged side-by-side by 48 hpf, in a process known as cardiac looping [56]. In the majority of *epn2*- and *atf7ip*-targeted embryos, cardiac looping defects were observed by 48 hpf, with the atrium and ventricle arranged more linearly along the anterior–posterior axis compared with control embryos (Fig 5E–H). The looping defects became more pronounced by 4 dpf such that the chambers were often linearly arranged, with small, compact ventricles (Fig 5I–K).

In support of an evolutionarily conserved role for *Atf7ip* in cardiac development, we observed structural defects in the alignment of the great vessels and ventricular morphology in myocardial-specific (*MHC-Cre*) *Atf7ip* knockout mice (S2A–C Fig). Additional cardiac developmental abnormalities observed in *Atf7ip* myocardial-specific knockout embryos included pericardial haemorrhage or oedema (n = 6), abnormal or hypoplastic right ventricle morphology (n = 5), double outlet right ventricle (n = 1), common ventricle (n = 1), peri-membranous ventricular septal defects (n = 5), and exencephaly (n = 3). These results indicate that our RF classifier has identified novel genes required for normal cardiac development.

### Cardiac development gene database

We developed a publicly accessible database, CDGD (Cardiac Development Gene Database; URL: http://130.88.96.175/), which contains information regarding the cardiac/non-cardiac status of all protein-coding genes within the mouse genome, derived either from published literature or our predictions. The database contains confidence scores indicating the predicted probabilities of the genes to be cardiac or non-cardiac. The database can be searched for known or predicted mouse genes using various identifiers such as gene name, MGI ID, Ensembl ID, and UniProt ID. Furthermore, users can retrieve lists of all cardiac and non-cardiac genes (both known and predicted) within the mouse genome or within specific chromosomal and genomic regions. All search results are downloadable as CSV files for further analysis.

### PPI networks of cardiac and non-cardiac genes

**Fig 6:**
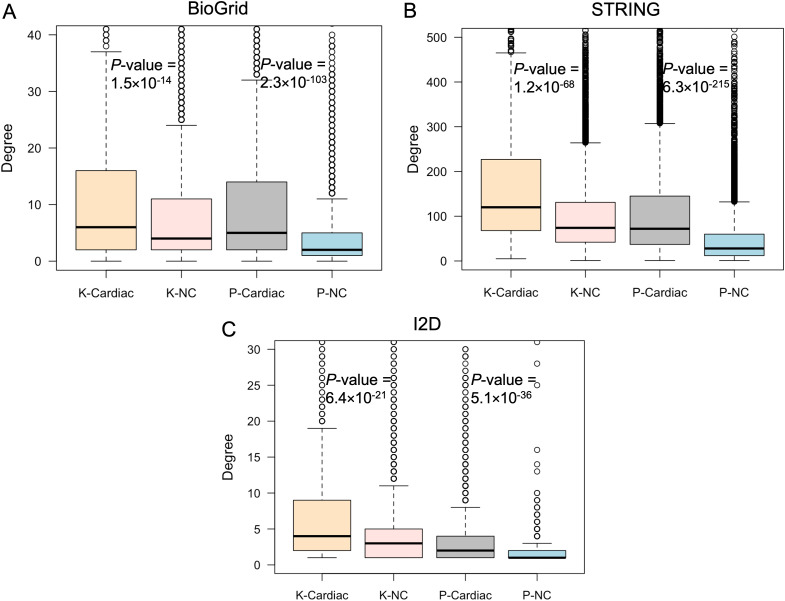
Degree of cardiac and non-cardiac proteins within the PPI networks sourced from BioGRID (A), STRING (B) and I2D (C) databases. Here, K-Cardiac represents the PPI network of known cardiac proteins; K-NC represents the PPI network of known non-cardiac proteins; P-Cardiac represents the PPI network of predicted cardiac proteins; and P-NC represents the PPI network of predicted non-cardiac proteins. *P*-values were determined using the Mann-Whitney U test.

Since we found protein interaction network features to be highly informative for our classifier development, we decided to compare the topology features of protein interaction networks involving proteins whose cardiac status was known and those networks involving proteins with predicted status. We utilised PPI databases BioGRID [57] and STRING [58] along with I2D to extract protein interaction data for the encoded proteins of both predicted and known genes. Invoking the “guilt by association” principle, STRING data has been previously utilised to successfully predict novel CHD genes based on interactions with known CHD proteins [16], therefore, inclusion of interaction data from STRING is therefore highly relevant to our cardiac gene predictions analysis. We excluded PPIs from the STRING database lacking experimental evidence or with confidence scores below 0.4. Based on the guilt-by-association principle, we would expect a greater number of interactions between known and predicted cardiac proteins. Our observations confirmed that known cardiac proteins exhibit more interactions with predicted cardiac proteins than with predicted non-cardiac proteins. This trend was consistently observed across all three PPI databases (S14 Table). Furthermore, we constructed four distinct PPI networks from each database: K-Cardiac (encompassing PPIs for all known cardiac proteins), K-NC (encompassing PPIs for all known non-cardiac proteins), P-Cardiac (encompassing PPIs for all predicted cardiac proteins), and P-NC (encompassing PPIs for all predicted non-cardiac proteins) networks. Using the “network analyzer” plugin of Cytoscape v3.9.1, we computed the network properties for these networks. Our analysis revealed that known cardiac proteins exhibited a higher number of interactions (higher degrees) compared to known non-cardiac proteins within their respective interaction networks, consistent across both BioGRID and STRING (Fig 5), and mirroring observations made with I2D. Furthermore, we found a similar trend with the PPI networks generated from our predicted cardiac proteins, which also displayed higher degrees compared to networks of predicted non-cardiac proteins. These trends remained consistent across all PPI databases, as confirmed by Mann-Whitney U statistical tests (Fig 6). Notably, many of the predicted cardiac proteins have fewer interactions compared to known cardiac proteins, yet they were still predicted to be associated with cardiac development. One reason for this could be that they are less studied, so many of their PPIs are not yet known.

### Developmental expression patterns of cardiac and non-cardiac genes

Genes involved in the same biological process are expected to exhibit similar expression patterns, consistent with the principle of “guilt by association” [59]. Accordingly, we hypothesised that genes in our predicted datasets would display similarities in developmental expression patterns (co-expression) to those of known genes within each gene class. To test this hypothesis, we analysed developmental gene expression profiles across four distinct categories of gene pairs: (1) predicted cardiac vs known cardiac, (2) predicted cardiac vs known non-cardiac, (3) predicted non-cardiac vs known cardiac, and (4) predicted non-cardiac vs known non-cardiac to test our hypothesis.

**Fig 7:**
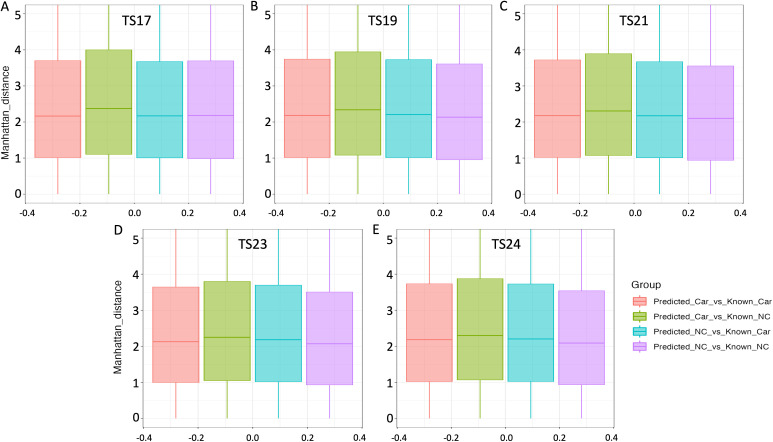
Measurements of co-expression. Differences in Manhattan distance across Theiler 17 (A), 19 (B), 21 (C), 23 (D) and 24 (E) stages in mice for four groups of gene pairs: (1) predicted cardiac vs known cardiac (Predicted_Car_vs_Known_Car), (2) predicted cardiac vs known non-cardiac (Predicted_Car_vs_Known_NC), (3) predicted non-cardiac vs known cardiac (Predicted_NC_vs_Known_Car), and (4) predicted non-cardiac vs known non-cardiac (Predicted_NC_vs_Known_NC). Here, “Manhattan_distance” indicates the level of co-expression between gene pairs. Larger Manhattan distances signify a greater dissimilarity in expression between two genes.

Our investigation drew upon RNA-Seq gene expression data sourced from the BGEE database [60]. We assessed the degree of developmental co-expression among each mouse gene pair within the above-mentioned gene groups across five pivotal Theiler stages (TS17, TS19, TS21, TS23 and TS24) of mouse development, employing the Manhattan distance method (Fig 7). In this context, larger Manhattan distances indicate a greater divergence in expression between two genes. Consistent with our expectations, we observed that predicted cardiac genes are more likely to exhibit similarities in developmental expression patterns with known cardiac genes in mouse (small distances and thus higher co-expression) throughout all Theiler stages when compared with both known non-cardiac genes and predicted non-cardiac genes, as confirmed by Kruskal-Wallis statistical tests (*P*-value < 0.05). Conversely, predicted non-cardiac genes demonstrated similar expression patterns with known non-cardiac genes, in comparison to known and predicted cardiac genes.

### Overlap with the “Unknome”

A recent study [61] investigated evolutionary conserved genes of unknown function to assess whether understudied genes play important biological roles. The authors termed the collection of genes lacking functional annotations the “unknome”. Experimental analysis of a subset of the unknome from *Drosophila* revealed that most poorly annotated genes did indeed have attributable biological functions, with clear phenotypes arising from mutations of these genes. We were therefore interested in determining whether our predicted gene datasets contained genes within the unknome and whether our predictions could serve as a further way to assist with functional annotations of understudied genes. We found that 74% of the *Drosophila* unknome genes analysed by Rocha et al., (2023) had orthologues within our predicted genes datasets, which include both cardiac and non-cardiac predictions. Given the substantial yield of informative functional data arising from experimental study of the unknome in *Drosophila*, we propose that similar experimental investigation of our predicted gene datasets will uncover novel biological associations. In particular, analysis of our predicted cardiac development gene dataset will be likely to identify pathways and molecules functioning in the process of heart development which are currently unknown.

### Comparison to new cardiac mouse knockouts

As noted above, experimental validation of our predictions is needed to confirm if our predicted cardiac gene dataset does indeed include genes newly found to play a role in cardiac development. To explore available experimental evidence, we conducted a search in the MGI database using the phenotype annotation “abnormal heart morphology” to identify mouse knockouts with cardiac defects reported after the development of our classifier. The aim was to obtain a set of genes that were not included in our training datasets but have since been experimentally validated as having roles in cardiac development. This search yielded a total of 69 genes (as of August 2025). To assess the accuracy of our classifier, we compared these recently published cardiac genes with our gene predictions and observed a significant overlap with our predicted cardiac genes, as illustrated in S15 Table. Notably, our classifier correctly predicted 78% (54 out of 69 genes) of these new cardiac knockouts to be associated with cardiac development, with false negatives tending to have lower confidence scores. The top 10 genes from this new dataset are listed in Table 5. This substantial alignment between our predictions and the new cardiac development genes underscores the efficacy of our classifier’s performance and validates the robustness of our approach in identifying genes involved in cardiac development.

**Table 5:** Top 10 recently published mouse cardiac genes and their prediction status by our Random Forest classifier.

Gene Symbol	Encoded protein and function	Predicted Class	Confidence
*Plvap*	Plasmalemma vesicle-associated protein; involves in the formation of the diaphragms that bridge endothelial fenestrae	Cardiac	0.97
*Smarca4*	Smarca4 protein; involves in transcriptional activation and repression	Cardiac	0.945
*Pfkm*	Phosphofructokinase, muscle; catalyses the phosphorylation of D-fructose 6-phosphate to fructose 1,6-bisphosphate by ATP	Cardiac	0.92
*Csf1*	Macrophage colony-stimulating factor 1; plays an essential role in the regulation of survival, proliferation and differentiation of hematopoietic precursor cells	Cardiac	0.895
*Thbs1*	Thrombospondin-1; mediates cell-to-cell and cell-to-matrix interactions	Cardiac	0.89
*Axl*	AXL receptor tyrosine kinase; transduces signals from the extracellular matrix into the cytoplasm by binding growth factor GAS6	Cardiac	0.89
*Cse1l*	Chromosome Segregation 1 Like; nuclear import and export of cargo proteins	Cardiac	0.88
*Ager*	Advanced glycosylation end product-specific receptor; senses endogenous stress signals with a broad ligand repertoire	Cardiac	0.875
*Pnpla2*	Patatin-like phospholipase domain-containing protein; catalyses the initial step in triglyceride hydrolysis in adipocyte and non-adipocyte lipid droplets	Cardiac	0.86
*Mmp2*	72 kDa type IV collagenase; involves in angiogenesis, tissue repair, tumor invasion, inflammation	Cardiac	0.86

### Comparison to human CHD genes

We cross-referenced our predicted cardiac development gene dataset with multiple published datasets of human CHD genes, aiming to identify which CHD genes are orthologues of our predicted cardiac genes. Several recent studies have reported genes associated with human CHDs [14,16,62–67], many of whose mouse orthologues were not included in our machine learning training datasets. We observed a significant overlap between our predictions and these newly identified CHD gene datasets (Tables 6 and S16). Overall, 76% of the newly identified CHD genes were orthologues of mouse genes that we predicted to be associated with cardiac development (hypergeometric test, *P-*value = 1.3 × 10^-17^), while the remaining genes were predicted to be non-cardiac. This substantial overlap with published CHD-causative genes strongly supports the biological validity of our predictions and highlights the predicted cardiac development genes as promising candidates for human CHD.

**Table 6:** Cardiac development gene prediction matches to human CHDs. We compared the human orthologues of our mouse cardiac development gene predictions with human CHD genes identified in literature spanning from 2017 to 2024 to assess our classifier’s effectiveness in identifying CHD genes. If our classifier predicts a CHD gene as associated with cardiac development, we consider this prediction an accurate outcome.

Reference (Confirmed CHD genes)	Number of CHD genes identified	Number of CHD genes not included in our ML training sets	Number of CHD genes we predicted to be involved in cardiac development	Percentage CHD genes we predicted to be involved in cardiac development
Jin SC et al. Contribution of rare inherited and de novo variants in 2,871 congenital heart disease probands. Nature Genetics. 2017;49(11):1593–601. [14]	**252**	**113**	**81**	**72%**
Ma L et al. The HDAC9-associated risk locus promotes coronary artery disease by governing TWIST1. PLoS Genetics. 2022;18(6):e1010261. [65]	**2**	**1**	**1**	**100%**
Cui M et al. Transcription factor NFYa controls cardiomyocyte metabolism and proliferation during mouse fetal heart development. Developmental Cell. 2023;58(24):2867–80. [62]	**1**	**1**	**1**	**100%**
Doering L et al. CRISPR-based knockout and base editing confirm the role of MYRF in heart development and congenital heart disease. Disease Models & Mechanisms. 2023;16(8):dmm049811.[63]	**5**	**4**	**3**	**75%**
Huttner IG et al. Loss of Sec-1 Family Domain-Containing 1 (scfd1) Causes Severe Cardiac Defects and Endoplasmic Reticulum Stress in Zebrafish. Journal of Cardiovascular Development and Disease. 2023;10(10):408. [64]	**1**	**1**	**1**	**100%**
Pan Y, et al. Whole-exome sequencing revealed novel genetic alterations in patients with tetralogy of Fallot. Translational Pediatrics. 2023;12(10):1835. [66]	**2**	**2**	**2**	**100%**
Zhao E et al. The expanded spectrum of human disease associated with GREB1L likely includes complex congenital heart disease. Prenatal Diagnosis. 2024. [67]	**1**	**1**	**1**	**100%**
Chhatwal K et al. Uncovering the Genetic Basis of Congenital Heart Disease: Recent Advancements and Implications for Clinical Management. CJC Pediatric and Congenital Heart Disease. 2023. [68]	**15**	**2**	**2**	**100%**
Yang A et al. CHDgene: A Curated Database for Congenital Heart Disease Genes. Circ Genom Precis Med. 2022;15:e003539. [69]	**187**	**84**	**72**	**86%**
**Reference (Predicted CHD genes)**	**Number of predicted CHD genes identified**	**Number of genes not included in our ML training sets**	**Number of CHD genes we predicted to be involved in cardiac development**	**Percentage CHD genes we predicted to be involved in cardiac development**
Xie Y et al. Network assisted analysis of de novo variants using protein-protein interaction information identified 46 candidate genes for congenital heart disease. PLoS Genetics. 2022;18(6):e1010252. **[**16]	**46**	**28**	**24**	**86%**
Nappi F. In-depth genomic analysis: the new challenge in congenital heart disease. International Journal of Molecular Sciences. 2024 Feb 1;25(3):1734. [70]	**18**	**11**	**10**	**91%**
Schroeder AM et al. Model system identification of novel congenital heart disease gene candidates: focus on RPL13. Human Molecular Genetics. 2019; 28(23):3954–69. [71]	**2**	**2**	**2**	**100%**
Sevim Bayrak C, et al. De novo variants in exomes of congenital heart disease patients identify risk genes and pathways. Genome Medicine. 2020;12:1–8. [72]	**23**	**14**	**12**	**86%**
Sierant, M, et al. Genomic analysis of 11,555 probands identifies 60 dominant congenital heart disease genes. Proceedings of the National Academy of Sciences. 2025; 122(13):e2420343122. [73]	**60**	**21**	**19**	**90%**
Kars ME, et al. Deciphering the digenic architecture of congenital heart disease using trio exome sequencing data. The American Journal of Human Genetics. 2025 112(3):583–98. [74]	**19**	**8**	**8**	**100%**
TOTAL (confirmed and predicted CHD genes)	**732**	**345**	**263**	**76%**

We also assessed a total of 25 genes categorised as “Green” PanelApp [75] genes from the “Familial non-syndromic congenital heart disease” clinical testing panel (https://panelapp.genomicsengland.co.uk). These were genes included on the Genomics England panel used to assess participants with CHD recruited to the UK100KGP cohort and are therefore known human CHD disease genes. Our classifier correctly identified 88% (22 out of 25) of these genes as known or predicted cardiac genes, while the remaining 3 genes were classified as known or predicted non-cardiac genes.

We then evaluated a dataset containing 79 novel candidate genes found to contain high impact, rare, *de novo* variants identified in individual human CHD cases within the UK100 KG cohort [76] (S16 Table). Of these, 47% were orthologues of predicted or known cardiac genes in our mouse datasets, while 20% were predicted non-cardiac genes. This demonstrates our classifier’s capability to recognise human CHD genes effectively. Table 7 displays the 10 highest-ranking candidate CHD genes, with the complete list available in S16 Table.

**Table 7:** Top 10 classifier predictions for novel candidate CHD genes identified from the UK100 KG cohort.

Gene Symbol	Encoded Protein and Function	Predicted Class	Confidence
*CSTF3*	Cleavage stimulation factor subunit 3; required for polyadenylation and 3’-end cleavage of mammalian pre-mRNAs	Cardiac	0.955
*UBR2*	E3 ubiquitin-protein ligase UBR2; plays a critical role in chromatin inactivation	Cardiac	0.955
*ERC1*	ELKS/Rab6-interacting/CAST family member 1; regulatory subunit of the IKK complex	Cardiac	0.935
*CBLB*	E3 ubiquitin-protein ligase CBL-B; promotes ubiquitin degradation by the proteasome	Cardiac	0.93
*PFKFB2*	6-phosphofructo-2-kinase/fructose-2,6-bisphosphatase 3; Catalyses both the synthesis and degradation of fructose 2,6-bisphosphate	Cardiac	0.87
*NR4A1*	Nuclear receptor subfamily 4 group A member 1; orphan nuclear receptor	Cardiac	0.865
*ABCF3*	ATP-binding cassette sub-family F member 3; displays an antiviral effect against flavivirus	Cardiac	0.86
*AC008755.1 (ZC3H4)*	Zinc finger CCCH domain-containing protein 4; RNA-binding protein that suppresses transcription of long non-coding RNAs	Cardiac	0.85
*PYGB*	Glycogen phosphorylase, brain form; regulates glycogen mobilization	Cardiac	0.84
*CDC5L*	Cell division cycle 5-like protein; DNA-binding protein involved in cell cycle control	Cardiac	0.805

## Discussion

Advances in genomic research methodologies have expanded access to genome sequencing for CHD patients. However, a significant challenge arising from genomic analysis is linking variation within a specific gene to CHD causation in an individual patient. Machine learning, with its ability to uncover patterns within datasets, presents a promising method for identifying CHD-related genes. In this study, we developed a Random Forest (RF) classifier to identify genes likely required for cardiac development. These genes may serve as promising candidates for causing CHDs when mutated.

Our RF classifier achieved an 81% accuracy in a 10-fold cross-validation analysis following feature selection. The classifier demonstrated an 88% accuracy in predicting the cardiac development association status of known non-cardiac genes not utilised during the classifier’s training. Additionally, the classifier accurately predicted the cardiac development status for 89% of cardiac reporter line genes. These results suggest that our classifier is not overfitted, as there is no drop in performance between training and testing. The high accuracy observed on these independent test datasets suggests that our classifier effectively discriminates between cardiac and non-cardiac genes. Furthermore, our predictions of the cardiac development status of all protein-coding genes in the mouse genome revealed that approximately 36% of genes may play a role in cardiac development, while 64% likely do not. In our training sets of genes with known cardiac development status, only 29% of the known phenotype genes have cardiac developmental defects, indicating that there are many cardiac development genes currently lacking experimental validation.

Subsequent examination of the protein network topology of the predicted cardiac and non-cardiac genes demonstrated that proteins within the predicted cardiac network exhibit a high degree of connectivity, aligning with our observation for the known cardiac genes. Additionally, the protein interaction networks for known cardiac genes and predicted cardiac genes have similar connectivity characteristics. Moreover, analysis of RNA-Seq gene expression data across development indicated that predicted cardiac genes have overlap with known cardiac genes in their developmental expression patterns. These findings support the hypothesis that the known and predicted cardiac genes jointly function in the process of cardiac development.

We found that among our top 15 most confident cardiac gene predictions, *Sp3* and *Qki* have been identified as critical for cardiac development in mice. *Sp3* null hearts show defective looping at E10.5 and severe cardiac malformations at E14.5, highlighting the essential role of *Sp3* in normal cardiac development, particularly in myocardial differentiation [51]. A recent study demonstrated that *Qki* gene is vital for cardiovascular development, especially in the formation of cardiac mesoderm [53]. *Qki* knockout disrupts the alternative splicing program associated with cardiac mesoderm and impairs myocyte formation. Pathogenic variants in the gene *DVL1* cause Robinow syndrome in humans, which frequently features ventricular septal defects as part of the phenotype [54]. Dvl-mediated planar cell polarity (PCP) signalling, involving *Dvl1* or *Dvl2*, is essential in the second heart field (SHF) lineage for proper OFT development and plays a key role in OFT lengthening during cardiac looping [55]. Furthermore, we have experimentally tested two other genes from our top 15 most confident predictions. Mouse and zebrafish mutant models confirm that mutation of the *Epn2* and *Atf7ip* genes disrupts cardiac development. However, it should be noted that the mutant mouse and zebrafish models discussed here are generally homozygous null models, and thus do not fully recapitulate the heterozygous missense variation commonly seen in human CHD.

A large proportion of our predicted genes are orthologues of genes within the *Drosophila* unknome [61], which comprises genes without a current functional annotation. Our predictions can therefore begin to provide suggested functions for these genes, which may be borne out by further experimental analysis. Furthermore, comparison of the human orthologues of our predicted mouse cardiac genes with newly discovered human CHD genes revealed a 78% overlap, demonstrating the potential for our predictions to reveal candidate disease genes. One gene identified from newly reported mouse knockout experiments, *Smarca4* [14], has also recently been discovered to harbour pathological variants in patients with non-syndromic cleft lip and/or cleft palate and cardiac outflow tract defects [77]. An additional case report of a patient with Coffin-Siris syndrome with a novel variant in *SMARCA4* describes severe congenital heart disease as an aspect of the phenotype [78].

**Fig 8:**
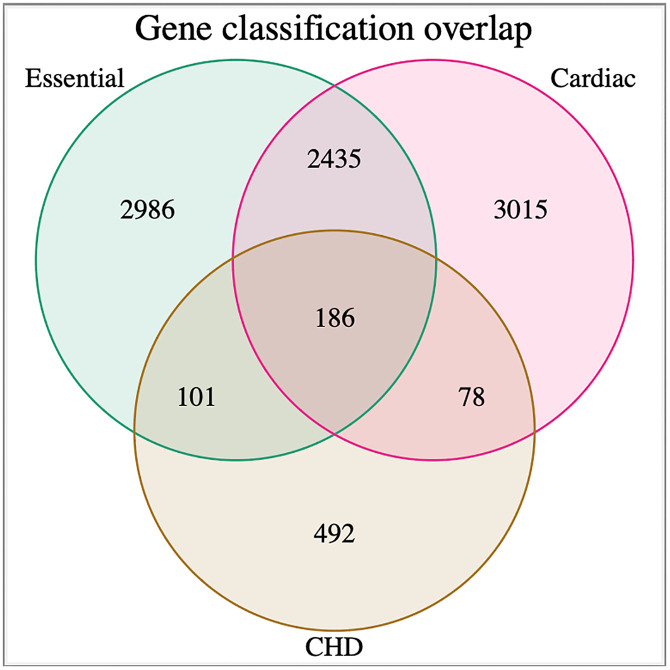
Measurements of overlap between predicted and known essential genes, predicted and known cardiac genes, and reported CHD genes. Essential gene list taken from Tian et al., 2018 [43]; cardiac gene list compiled from S1 and S15 Tables, CHD gene list compiled from S16 Table.

Our predicted cardiac genes may not fully overlap with CHD genes because our classifier was trained to identify genes involved in cardiac development, not on CHD gene features. Additionally, it is likely that some cardiac development genes will perform essential functions, and deleterious changes in those genes will cause embryonic lethality. Many of our known cardiac training set genes have embryonic lethal knockout phenotypes, confirming the essentiality of these genes. Consequently, individuals harbouring deleterious variants in these genes may not survive development, and therefore these genes would not be identified from CHD patient cohorts. As greater numbers of CHD genes are identified, the overlap between our predicted cardiac gene dataset and CHD genes may increase (Fig 8). Nevertheless, our classifier identified large numbers of candidate genes that can be examined through future experimental analysis as potential causative genes for CHD.

Our ML approach offers a rapid, cost-effective, and complementary means of identifying cardiac developmental genes, serving as a valuable complement to experimental methods. Through a single analysis, our approach yielded cardiac predictions for the entire genome. The ranked list of genome wide cardiac developmental gene predictions, available in our publicly accessible database, should be of value to researchers and clinicians investigating genetic causes of CHDs, and could serve as a complement to the PanelApp traffic-light system [75]. By enabling prioritisation of genes during the evaluation of CHD patient sequence data, this resource has the potential to accelerate genetic diagnoses, thereby facilitating more informed treatment and disease management strategies for individuals affected by CHD. Further investigation of our predicted cardiac genes should elucidate their roles in cardiac development and reveal potential clinical associations with CHD.

## Materials and methods

### Ethics statement

Mouse colonies were maintained at the Biological Service Facility at the University of Manchester, UK, according to Home Office requirements and with local ethical approval from the University of Manchester Animal Welfare and Ethical Review Body (project licence number PP3720525). All experiments involving live zebrafish (*Danio rerio*) were carried out in compliance with the Seattle Children’s Research Institute’s Institutional Animal Care and Use Committee guidelines (animal protocol number IACUC00124, approved 17 May 2024 –16 May 2027).

### Cardiac and non-cardiac mouse gene datasets

The MGI database was used to compile a dataset encompassing all mouse genes. To identify genes pertinent to cardiac development, we relied on information from the mouse knockout literature. This study included only null alleles of mouse genes, characterised by known phenotypes resulting from single gene knockout experiments (targeted deletions). Mouse genes were categorised as either cardiac or non-cardiac based on the mutant mouse phenotype data retrieved from the MGI database (as of June 17, 2021). We defined the phenotype of a knockout mouse as “cardiac” if the gene was known to be involved in cardiac development. These genes can potentially cause CHDs when mutated. Mouse knockouts with phenotypes known not to be associated with cardiac development were marked as “non-cardiac”. Some entries in these knockout datasets were ambiguous, annotated as both cardiac and non-cardiac in MGI. To resolve this ambiguity, we manually cross-referenced the phenotypes of these overlapping entries with published literature and included each gene in only a single category consistent with published phenotype descriptions. Mouse genes that were not labelled as either cardiac or non-cardiac were categorised as genes with “unknown” cardiac association status. Our datasets were limited to protein-coding genes only. We identified the encoded proteins for each mouse gene using the UniProt [79] (release 2021_3) database, selecting only the longest protein isoform for analysis per gene. Additionally, we obtained the Ensembl [80] gene identifier and UniGene [81,82] expression cluster identifier mapping for each MGI gene symbol.

### Feature collation

In machine learning, the individual pieces of information inputted into a classifier are termed “features”. These features are utilised by the classifier to recognise patterns that enable the prediction of whether a novel gene possesses similarities with genes within a specified training set. For our study, we assembled various features derived from gene and protein sequences, reflecting diverse aspects of mouse biology to distinguish between cardiac and non-cardiac phenotypes. These features encompass characteristics such as sequence properties, protein localisation and interaction details, developmental expression data, and gene ontology annotations. Computation of features such as “gene length”, “GC content”, “transcript count”, “exon count”, “exon length”, and “intron length” was performed using data extracted from the Ensembl database (release 103 of Mus musculus genes) through the Ensembl BioMart [83] data mining tool. Gene expression data, measured as transcripts per million (TPM), were sourced from the UniGene database for 13 embryonic developmental stages. Additionally, RNA-seq gene expression data spanning 8 tissue types (heart tissues of eight-week-old mice, fibroblast tissues of eight-week-old mice, multi-cell lifecycle, zygote, Ths10 epiblast, thymus tissues of eight-week-old mice, adipose tissues of twenty-four-week-old mice and stem cell) were obtained from the BGEE [60] v15.2 database. Protein characteristics including length, molecular weight, and amino acid composition were calculated using the Pepstats [84] program, with subcellular localisation features obtained from UniProt. Evolutionary age, signal peptides, transmembrane domains, and subcellular locations were acquired from Ensembl, SignalP [85], and UniProt. Mouse PPI data were sourced from the I2D [33] v2.3 database, and various properties of PPI networks were computed using the “network analyzer” plugin of Cytoscape [86] v3.9.1 and the Hubba web-based service. PPI data were also obtained from the BioGRID [57] v4.4 and STRING [58] v12.0 databases to conduct the PPI network analyses. Prior to network analyses, self-loops and duplicated edges were removed. Gene ontology (GO) terms were retrieved using the “Functional Annotation” tool of the DAVID [36] v2021q4 web-based application. Chromosome location data for all protein-coding mouse genes were obtained from Ensembl. Detailed explanations of these compiled features have already been provided in previous studies [26,42,43]. Furthermore, gene-level attributes, including gene constraint metrics such as probability of loss-of-function score (pLoF) and probability of being loss-of-function intolerant score (pLI), were obtained as features from the gnomAD v2.1.1 database [87].

### Machine learning classifiers

In this study, we developed classifiers using Random Forest (RF), Gradient Boosted Trees (GBT), J48 decision trees and Support Vector Machine (SVM) algorithms, incorporating a range of features to identify genes that are highly likely to play a role in cardiac development and may serve as promising candidates for CHDs when mutated. The implementation of these classifiers was carried out using WEKA v3.8.6, an open-source machine learning tool, alongside R [88]. To address the imbalanced classification issue, wherein a bias toward the larger gene group could occur, balanced training datasets were generated by employing random subsampling with no replacement [89] to ensure equal representation of cardiac and non-cardiac genes. Additionally, adjustments were made to refine features by replacing missing values with the respective feature mean values. Employing the 10-fold cross-validation technique on a dataset comprising both cardiac and non-cardiac mouse genes facilitated the prevention of overfitting; a classifier overfits when its prediction accuracy is high on the training dataset but poor on the test dataset. This approach entailed random partitioning of the training dataset into 10 equal subsets, with nine used for classifier training and one for testing. To classify a new gene, the features of the gene are evaluated using each of the decision trees within our Random Forest classifier. Each tree provides a class prediction or “vote,” and the class with the highest number of votes is chosen as the class to which the gene belongs. Our classifier produces a probability score that reflects the confidence level associated with prediction outcomes. This probability score is derived by averaging all predictions generated by decision trees within the Random Forest ensemble. Separate test datasets were generated from mouse genes not utilised in classifier training; evaluation of our classifier performance was assessed by estimating the proportion of accurately predicted genes within these test datasets.

### Feature selection

The correctness of classification depends significantly on the quality of the input features used in developing the classifier. Not all features within the training dataset are beneficial; irrelevant or redundant features can introduce noise and lead to overfitting. Feature selection is, therefore, a critical step in developing an accurate and efficient classifier, particularly when dealing with high-dimensional datasets containing numerous features, as it helps mitigate the effects of irrelevant and redundant features.

In this study, feature selection was performed using the Information Gain filter method implemented in WEKA, a widely used approach in machine learning for identifying the most relevant features for classification. This method was chosen due to its simplicity, and effectiveness in identifying informative features. Information Gain is based on entropy, a measure of uncertainty or impurity from information theory, making it particularly well-suited for classification problems with categorical target variables. This method evaluates the relevance of each feature by quantifying how much information about the target variable can be gained by knowing the value of the feature [90]. Features with higher information gain values are more informative, as they contribute more to reducing uncertainty about the target variable.

The choice of Information Gain for this study is supported by its advantages over other feature selection methods. First, it is computationally efficient, which is important given the large number of features in this dataset. Second, it provides a clear, interpretable ranking of features, allowing a straightforward prioritisation of the most important ones. By selecting the most informative features for distinguishing between cardiac and non-cardiac genes using Information Gain, this study aims to reduce dimensionality, minimise noise, and enhance the generalizability of the classifier. This approach helps address challenges such as overfitting and dataset-specific biases, ultimately leading to a more robust and interpretable model.

### Evaluation metrices

The performance of our machine learning classifier was evaluated using several metrics, including recall, precision and accuracy, defined by Equations (1)–(3), where *TP*, *TN*, *FP*, and *FN* represent the number of true positives (i.e., correctly identified cardiac genes), true negatives (i.e., correctly identified non-cardiac genes), false positives (i.e., incorrectly identified non-cardiac genes), and false negatives (i.e., incorrectly identified cardiac genes), respectively. Additionally, the area values of receiver operating characteristic curve (AUC) and precision-recall curve (PRC) and confusion matrix were also considered for classifier evaluation. The ROC area provides a measure of overall classifier performance, while the PRC area evaluates the classifier’s performance in identifying samples from individual groups.


$$Recall=\frac{TP}{TP+FN}$$ (1)

$$Precision=\frac{TP}{TP+FP}$$ (2)

$$Accuracy=\frac{TP+TN}{TP+TN+FP+FN}$$ (3)

### Gene co-expression analysis

To examine if cardiac genes have similarities in developmental expression patterns, RNA-Seq gene expression data were obtained from the latest version of BGEE (release 15) which contains mouse expression data compiled from 566 libraries. We retrieved tissue-based RNA-Seq expression data represented as transcripts per million (TPM) for Theiler developmental stages 17, 19, 21, 23, and 24. These TPM values were transformed into corresponding log values using Equation 4 to measure co-expression between every gene pair.


$$L_{TPM}={log}_{\text{2}}(TPM+\text{1})$$ (4)

Gene pairs were categorised into four groups: (1) predicted cardiac versus known cardiac, (2) predicted cardiac versus known non-cardiac, (3) predicted non-cardiac versus known cardiac, and (4) predicted non-cardiac versus known non-cardiac. We calculated Manhattan distances to generate numerical scores representing gene co-expression for each category. Distances were computed for every gene pair across each Theiler stage to assess developmental expression patterns. For two mouse genes, *a* and *b*, with expression values, the Manhattan distance was calculated using Equation 5, based on log-transformed TPM data (Equation 4). Lower distance values indicate higher co-expression between genes.


ManDis(a,b)=|a−b| (5)

### Unknome comparison

We retrieved mouse orthologues for a list of 358 *Drosophila* genes using the DRSC Integrative Ortholog Prediction Tool (DIOPT) [91]. The genes that lacked known functions formed the “unknome” [61].

### RT-PCR verification of most confidently predicted cardiac genes

Embryonic and adult heart tissues were isolated from wild type 129S5/SvEvBrd mice and homogenised in Trizol. Total RNA was extracted, and complementary DNA (cDNA) was synthesised using High-Capacity cDNA Reverse Transcription Kit (Applied Biosystems). RT-PCR was conducted using PCR-Biosciences Ultra Mix Red polymerase with primers (S17 Table) designed to amplify top predicted cardiac genes. PCR products were analysed by agarose gel electrophoresis and visualised under a UV transilluminator. 18S rRNA expression was used as a positive control for confirming the presence of cDNA template in all reactions.

### Mouse conditional knockout analysis

To generate cardiac-specific Atf7ip knockout mice, *Tg(Myh6-cre)*^*2182Mds/J*^ mice were crossed to *Atf7ip*^*fl/fl*^ mice [92]. Genotyping was performed as described [92]. Mouse colonies were maintained at the Biological Service Facility at the University of Manchester, UK. Mice were euthanised using a Schedule 1 method following UK Home Office regulations. Embryos were dissected in PBS and imaged on a Leica MZ6 microscope with DFC420 camera. For histology embryos were fixed, embedded in paraffin, sectioned and stained with haemotoxylin and eosin as previously described [93].

### Zebrafish husbandry and CRISPR experiments

All experiments involving live zebrafish (*Danio rerio*) were carried out in compliance with Seattle Children’s Research Institute’s Institutional Animal Care and Use Committee guidelines. Zebrafish were raised and staged as previously described [94]. Time indicated as hpf or dpf refers to hours or days post-fertilization at 28.5°C.

The wild-type stock and genetic background used was AB. The *Tg(myl7:EGFP)*^*twu34*^ line has been previously described [95]. For fish stock maintenance, eggs were collected from 20–30 min spawning periods and raised in Petri dishes in ICS water [96] in a dark 28.5°C incubator, up to 5 dpf. After 5 dpf, the fish were maintained on a recirculating water system (Aquaneering) under a 14 h on, 10 h off light cycle. From 6–30 dpf, the fish were raised in 2.8 L tanks with a density of no more than 50 fish per tank and were fed a standard diet of paramecia (Carolina) one time per day and Zeigler AP100 dry larval diet two times per day. From 30 dpf onwards, the fish were raised in 6 L tanks with a density of no more than 50 fish per tank and were fed a standard diet of Artemia nauplii (Brine Shrimp Direct) and Zeigler adult zebrafish feed, each two times per day.

The sequences for the oligonucleotides (Table 8) used to synthesize the single-guide RNAs for CRISPR targeting were taken from the genome-scale lookup table provided by [53]. For a negative control 4-guide set, we used the “Genetic Screen Scramble1 Control” guides of [53]. sgRNAs were synthesized as described [53]. For phenotype analysis, 2 μL of a 4-guide cocktail of sgRNA at 2 μg/μL was combined with 2 μL of Cas9 protein (IDT Alt-R S.p. Cas9 Nuclease V3) at 10 μM (diluted as in [53]) and incubated at 37°C for 5 minutes. One microliter of phenol red injection solution (0.1% phenol red and 0.2M KCl in water) was added to generate the working solution for injections. One-cell stage embryos, collected from *Tg(myl7:EGFP)twu34* fish, were injected in the yolk with one nanoliter of the RNP 943 working solution. Following 1-cell stage injections, embryos were allowed to develop further and cardiac phenotype was assessed in live embryos using GFP expression. For imaging, representative live embryos were anaesthetized in tricaine (Sigma) and then transferred to 2.5% methyl cellulose (Sigma) in ICS water. To facilitate imaging of the GFP-expressing hearts, melanin formation was inhibited in some clutches by raising them from approximately 22 hpf in 0.003% *N*-phenylthiourea (Sigma) in ICS water. Both brightfield and epi-fluorescent images were captured with an Olympus DP74 camera mounted on an Olympus SZX16 stereomicroscope using cellSens Dimension imaging software. Merged brightfield and GFP images were made in Adobe Photoshop.

**Table 8:** Oligonucleotides for sgRNA synthesis for G0 CRISPANT analysis.

Oligo Name	Sequence 5’ - 3’
Scaffold_sgRNA	AAAAGCACCGACTCGGTGCCACTTTTTCAAGTTGATAACGGACTAGCCTTATTTTAAC TTGCTATTTCTAGCTCTAAAAC
ScreenScramble1_sg1	TAATACGACTCACTATAGGCTTGTGTCCCAACAGGCTGTTTTAGAGCTAGAAATAGC
ScreenScramble1_sg2	TAATACGACTCACTATAGGAAAGCTACGTCCAATTCTGTTTTAGAGCTAGAAATAGC
ScreenScramble1_sg3	TAATACGACTCACTATAGGTTTAGCGTGACAGCCGCAGTTTTAGAGCTAGAAATAGC
ScreenScramble1_sg4	TAATACGACTCACTATAGGTTAGATAAGGCATCCCGCGTTTTAGAGCTAGAAATAGC
epn2_sg1	TAATACGACTCACTATAGGGAACTGGAGGGCCCCCATGTTTTAGAGCTAGAAATAGC
epn2_sg2	TAATACGACTCACTATAGGCAACAATCAGATCGGCTTGTTTTAGAGCTAGAAATAGC
epn2_sg3	TAATACGACTCACTATAGGTCAGTCCCTTGATTGGCAGTTTTAGAGCTAGAAATAGC
epn2_sg4	TAATACGACTCACTATAGGAGCAGGCATTGGGCCCCAGTTTTAGAGCTAGAAATAGC
atf7ip_sg1	TAATACGACTCACTATAGGACGGCACTCATACCGAGAGTTTTAGAGCTAGAAATAGC
atf7ip_sg2	TAATACGACTCACTATAGGTAAAGAGAACATTGTCCGGTTTTAGAGCTAGAAATAGC
atf7ip_sg3	TAATACGACTCACTATAGGAAGTTGGCGTAGCCGGCGGTTTTAGAGCTAGAAATAGC
atf7ip_sg4	TAATACGACTCACTATAGGACAGCCTGGGCGTCCTAAGTTTTAGAGCTAGAAATAGC

### Statistics

All statistical analyses were performed using SPSS v27 and R. Feature significance was evaluated using the non-parametric Mann-Whitney U test, while differences in feature frequencies between cardiac and non-cardiac datasets were assessed with the Chi-squared (χ²) tests. *P*-values were adjusted for multiple comparisons using the Bonferroni correction [97]. Zebrafish CRISPR experiment data were analysed and visualised using GraphPad Prism 10.5, with statistical significance determined by one-way ANOVA followed by Dunnett’s correction for multiple comparisons. Enrichment of overlap between predicted cardiac development genes and human CHD genes was assessed using a hypergeometric test, which calculates the exact probability of observing such overlap by chance in discrete gene datasets.

## Supporting information

S1 Table Cardiac gene dataset. (XLSX)

S2 Table Non-cardiac gene dataset. (XLSX)

S3 Table Top 10 GO terms for mouse cardiac development genes related to biological processes, identified using the DAVID Functional Annotation tool. (XLSX)

S4 Table Top 10 GO terms for mouse non-cardiac genes related to biological processes, identified using the DAVID Functional Annotation tool. (XLSX)

S5 Table Top 10 GO terms for mouse cardiac development genes related to cellular component, identified using the DAVID Functional Annotation tool. (XLSX)

S6 Table Top 10 GO terms for mouse non-cardiac development genes related to cellular component, identified using the DAVID Functional Annotation tool. (XLSX)

S7 Table Test dataset 1–5331 Non-cardiac genes not included in the training dataset. (XLSX)

S8 Table Test dataset 2–966 mouse reporter lines mutants. (XLSX)

S9 Table Features selected using the information gain method. Features are sorted in descending order with respect to corresponding information gain value.
(XLSX)

S10 Table 10-fold cross validation performance of 5 different Random Forest classifiers. (XLSX)

S11 Table Performance of the Random Forest, Gradient Boosted Tree (GBT), J48 decision tree and Support Vector Machine (SVM) classifiers on Training dataset 1. (XLSX)

S12 Table Test dataset 3 - Remaining 12,375 genes for prediction. (XLSX)

S13 Table Prediction results on the entire mouse genome (Genes within Test dataset 1 and 2 are not included). Genes are ranked according to the confidence score.
(XLSX)

S14 Table Known cardiac proteins interact more frequently with the predicted cardiac proteins compared to predicted non-cardiac proteins. (XLSX)

S15 Table New Cardiac Mouse knockouts obtained from MGI (accessed March 21, 2024). (XLSX)

S16 Table Datasets of human CHD genes and prediction status of their mouse orthologues. (XLSX)

S17 Table RT-PCR primers - Top 15 genes. (XLSX)

S18 Table Provided as a Word file, lists the features collected and their corresponding data types. (DOCX)

S1 Fig Verification of expression of the 15 most confidently predicted cardiac genes through RT-PCR. Analysis was performed on cDNA isolated at different stages, including (A) E10.5 mouse whole embryo, (B) E15.5 mouse embryonic heart, and (C) adult mouse heart samples. Genes examined include *Epn2, Gne, Sp3, Cdk2, Bmi1, Ccne1, Dvl1, Qki, Dpysl2, Cenpc1, Gna12, Dnmt1, Atf7ip, Vps26a*, and *Enah*. The expression of 18S cDNA was used as a positive control for the presence of a cDNA template. DNA ladder 100 bp (Bioline).
(TIFF)

S2 Fig Embryonic cardiac phenotypes and reproductive outcomes in myocardial-specific *Atf7ip* knockout mice. Representative images of E16.5 embryonic hearts and corresponding H&E-stained heart sections. (A–C) Whole-heart images from control and *Atf7ip* myocardial-specific knockout embryos. (A′–C′) H&E-stained sections of the same hearts. Images show cardiac structural abnormalities, including double outlet right ventricle (DORV) and single ventricular morphology (magnification x2.5).
(PDF)
