# Supplementary material for: A machine learning classifier to identify and prioritise genes associated with murine cardiac development
Source: PLoS Genet. 2026 Feb 10;22(2):e1011489. doi: 10.1371/journal.pgen.1011489 (PMC12919933; doi:10.1371/journal.pgen.1011489)
Supplement: S18 Table — (DOCX) [file pgen.1011489.s018.docx]

**Table S18. Defining the Column Headings of Tables** **S1 and S2**

Tables S1 and S2 contain comprehensive data on gene and protein identifiers of cardiac and non-cardiac genes, along with detailed information on 127 specific features that were analysed. The followings are the definitions of the column headings used in these tables:

| **Column heading** | **Details** | **Feature type** | **Data type** |
| --- | --- | --- | --- |
| **Gene_name** | Standard abbreviation for the mouse gene name | Identifier | Alphanumeric character string |
| **MGI_ID** | Unique identifier for each gene in in the Mouse Genome Informatics database |  | Alphanumeric character string |
| **Ensembl_ID** | An Ensembl stable mouse gene ID |  | Alphanumeric character string; begins with ENS for Ensembl and a G for gene |
| **Longest_TrasncriptID** | An Ensembl stable mouse transcript ID |  | alphanumeric character string; begins with ENS for Ensembl and a T for transcript |
| **UniProt_ID** | Unique identifier for the protein in UniProt |  | Alphanumeric character string |
| **UniProt_Accession** | UniProt accession number for the protein |  | Alphanumeric character string |
| **Chromosome** | Chromosomal position where the gene is located | Chromosome location | Numerical |
| **Gene_Length** | Length of the gene | Gene sequence features | Numerical |
| **Transcript_count** | Total number of distinct RNA transcripts that are annotated for a particular gene |  | Numerical |
| **ExonCount** | Total number of distinct exons in the longest transcript of the gene |  | Numerical |
| **ExonLength** | Total length of exons |  | Numerical |
| **IntronLength** | Total length of introns |  | Numerical |
| **ProteinLength** | Length of the longest encoded protein sequence | Protein sequence features | Numerical |
| **MW** | Molecular weight: the total mass of the protein sequence |  | Numerical |
| **Aliphatic** | Proportion of aliphatic amino acids in the protein sequence |  | Numerical |
| **Aromatic** | Proportion of aromatic amino acids in the protein sequence |  | Numerical |
| **Non-polar** | Proportion of non-polar amino acids in the protein sequence |  | Numerical |
| **Polar** | Proportion of polar amino acids in the protein sequence |  | Numerical |
| **Charged** | Proportion of charged amino acids in the protein sequence |  | Numerical |
| **Basic** | Proportion of basic amino acids in the protein sequence |  | Numerical |
| **Acidic** | Proportion of acidic amino acids in the protein sequence |  | Numerical |
| **A** | Proportion of Alanine in the protein sequence |  | Numerical |
| **C** | Proportion of Cysteine in the protein sequence |  | Numerical |
| **D** | Proportion of Aspartic Acid in the protein sequence |  | Numerical |
| **E** | Proportion of Glutamic Acid in the protein sequence |  | Numerical |
| **F** | Proportion of Phenylalanine in the protein sequence |  | Numerical |
| **G** | Proportion of Glycine in the protein sequence |  | Numerical |
| **H** | Proportion of Histidine in the protein sequence |  | Numerical |
| **I** | Proportion of Isoleucine in the protein sequence |  | Numerical |
| **K** | Proportion of Lysine in the protein sequence |  | Numerical |
| **L** | Proportion of Leucine in the protein sequence |  | Numerical |
| **M** | Proportion of Methionine in the protein sequence |  | Numerical |
| **N** | Proportion of Asparagine in the protein sequence |  | Numerical |
| **P** | Proportion of Proline in the protein sequence |  | Numerical |
| **Q** | Proportion of Glutamine in the protein sequence |  | Numerical |
| **R** | Proportion of Arginine in the protein sequence |  | Numerical |
| **S** | Proportion of Serine in the protein sequence |  | Numerical |
| **T** | Proportion of Threonine in the protein sequence |  | Numerical |
| **V** | Proportion of Valine in the protein sequence |  | Numerical |
| **W** | Proportion of Tryptophan in the protein sequence |  | Numerical |
| **Y** | Proportion of Tyrosine in the protein sequence |  | Numerical |
| **GlycoProtein** | Indicates the presence or absence of glycosylation in the protein sequence | Post-translational Modifications | Boolean (0= is not glycosylated, 1 = is glycosylated) |
| **Phosphoprotein** | Indicates whether the protein is annotated as phosphoprotein or not |  | Boolean (0= is not phosphoprotein, 1 = is phosphoprotein) |
| **Acetylation** | Indicates proteins with acetylation modifications |  | Boolean (0= is not acetylated, 1 = is acetylated) |
| **Transcription** | Indicates proteins involved in transcription |  | Boolean (0= is not a transcription factor, 1 = is a transcription factor) |
| **SignalPeptide** | Indicates the presence or absence of signal peptide in the protein sequence | Protein sequence feature | Boolean (0= no signal peptide site, 1 = has signal peptide site) |
| **ec1** | Indicates whether the protein is annotated as an Oxidoreductase or not | Enzyme class | Boolean (0= not Oxidoreductase, 1 = Oxidoreductase) |
| **ec2** | Indicates whether the protein is annotated as Transferase or not |  | Boolean (0= not Transferase, 1 = Transferase) |
| **ec3** | Indicates whether the protein is annotated as Hydrolase or not |  | Boolean (0= not Hydrolase, 1 = Hydrolase) |
| **ec4** | Indicates whether the protein is annotated as Lyase or not |  | Boolean (0= not Lyase, 1 = Lyase) |
| **ec5** | Indicates whether the protein is annotated as Isomerase or not |  | Boolean (0= not Isomerase, 1 = Isomerase) |
| **ec6** | Indicates whether the protein is annotated as Ligase or not |  | Boolean (0= not Ligase, 1 = Ligase) |
| **ec7** | Indicates whether the protein is annotated as translocase or not |  | Boolean (0= not translocase, 1 = translocase) |
| **Nucleus_UniProt** | Indicates whether the protein is localised in the nucleus or not | Subcellular Localisation | Boolean (0= not in the nucleus, 1 = in the nucleus) |
| **Cytoplasm_UniProt** | Indicates whether the protein is localised in the cytoplasm or not |  | Boolean (0= not in the cytoplasm, 1 = in the cytoplasm) |
| **Plasma_UniProt** | Indicates whether the protein is localised in the plasma membrane or not |  | Boolean (0= not in the plasma membrane, 1 = in the plasma membrane) |
| **Membrane_UniProt** | Indicates whether the protein is localised in the non-plasma membrane or not |  | Boolean (0= not in the membrane, 1 = in the membrane) |
| **Extracellular_UniProt** | Indicates whether the protein is localised in the extracellular region or not |  | Boolean (0= not in the extracellular region, 1 = in the extracellular region) |
| **Mitochondrion_UniProt** | Indicates whether the protein is localised in the mitochondria or not |  | Boolean (0= not in the mitochondria, 1 = in the mitochondria) |
| **ER_UniProt** | Indicates whether the protein is localised in the endoplasmic reticulum (ER) or not |  | Boolean (0= not in the ER, 1 = in the ER) |
| **Golgi_UniProt** | Indicates whether the protein is localised in the Golgi apparatus or not |  | Boolean (0= not in the Golgi apparatus, 1 = in the Golgi apparatus) |
| **Lysosome_UniProt** | Indicates whether the protein is localised in the lysosome or not |  | Boolean (0= not in the lysosome, 1 = in the lysosome) |
| **Peroxisome_UniProt** | Indicates whether the protein is localised in the Peroxisome or not |  | Boolean (0= not in the Peroxisome, 1 = in the Peroxisome) |
| **CellJunction_Uniprot** | Indicates whether the protein is localised in the cell junction or not |  | Boolean (0= not in the cell junction, 1 = in the cell junction) |
| **CellProjection_Uniprot** | Indicates whether the protein is localised in the cell projection or not |  | Boolean (0= not in the cell projection, 1 = in the cell projection) |
| **Transmembrane_Count** | Indicates the number of predicted transmembrane helices or segments within the protein sequence | Protein sequence feature | Numeric |
| **AverageShortestPathLength** | Length of average shortest path in the protein interaction network | Protein-protein interaction network features | Numeric |
| **BetweennessCentrality** | Betweenness centrality value in the protein interaction network |  | Numeric |
| **ClosenessCentrality** | Closeness centrality value in the protein interaction network |  | Numeric |
| **ClusteringCoefficient** | Clustering coefficient value in the protein interaction network |  | Numeric |
| **Degree** | Degree in the protein interaction network |  | Numeric |
| **TopologicalCoefficient** | Topological coefficient value in the protein interaction network |  | Numeric |
| **MCC** | Length of average shortest path in the protein interaction network |  | Numeric |
| **DMNC** | Density of Maximum Neighbourhood Component in the protein interaction network |  | Numeric |
| **MNC** | Maximum Neighbourhood Component in the protein interaction network |  | Numeric |
| **EPC** | Edge Percolation Component in the protein interaction network |  | Numeric |
| **BottleNeck** | BottleNeck value in the protein interaction network |  | Numeric |
| **Eigenvector** | **Eigenvector** centrality value in the protein interaction network |  | Numeric |
| **LAC** | Local Average Connectivity in the protein interaction network. |  | Numeric |
| **Oocyte(Transcript/Million)** | Expression during the oocyte stage of mouse development | UniGene expression | Numeric |
| **Unfertilized_Ovum(Transcript/Million)** | Expression at the ovum stage of mouse development |  | Numeric |
| **Zygote(Transcript/Million)** | Expression during the zygote stage of mouse development |  | Numeric |
| **Cleavage(Transcript/Million)** | Expression during the cleavage stage of mouse development |  | Numeric |
| **Morula(Transcript/Million)** | Expression during the morula stage of mouse development |  | Numeric |
| **Blastocyst(Transcript/Million)** | Expression during the blastocyst stage of mouse development |  | Numeric |
| **Egg_Cylinder(Transcript/Million)** | Expression during the egg-cylinder stage of mouse development |  | Numeric |
| **Gastrula(Transcript/Million)** | Expression during the gastrula stage of mouse development |  | Numeric |
| **Organogenesis(Transcript/Million)** | Expression during the organogenesis stage of mouse development |  | Numeric |
| **Fetus(Transcript/Million)** | Expression during the fetus stage of mouse development |  | Numeric |
| **Neonate(Transcript/Million)** | Expression during the neonate stage of mouse development |  | Numeric |
| **Juveline(Transcript/Million)** | Expression during the juvenile stage of mouse development |  | Numeric |
| **Adult(Transcript/Million)** | Expression during the adult stage of mouse development |  | Numeric |
| **Age** | Evolutionary ages of the gene | Ensembl gene age | Numeric |
| **EightWeek_Heart** | RNA-Seq gene expression data for the heart tissue at the eight-week stage in mice | RNA-Seq gene expression | Numeric |
| **EightWeek_Fibroblast** | RNA-Seq gene expression data for the fibroblast tissue at the eight-week stage in mice |  | Numeric |
| **MultCell_Lifecyle** | RNA-Seq gene expression data that spans multiple stages of mouse development |  | Numeric |
| **Ths10_Epiblast** | RNA-Seq gene expression data to the epiblast at Theiler Stage 10 (Ths10) in mice |  | Numeric |
| **Zygote** | RNA-Seq gene expression data to the zygote stage in mice |  | Numeric |
| **EightWeek_Thymus** | RNA-Seq gene expression data for the **Thymus** tissue at the eight-week stage in mice |  | Numeric |
| **24Week_Adipose** | RNA-Seq gene expression data for the **Adipose** tissue at the twentyfour-week stage in mice |  | Numeric |
| **StemCell** | RNA-Seq gene expression data specific to stem cells in mice |  | Numeric |
| **GO:0007507** | Indicates gene’s involvement in heart development | Gene Ontology (GO) terms | Boolean (0= not in the cell projection, 1 = in the cell projection) |
| **GO:0003007** | Indicates gene’s involvement in heart morphogenesis |  | Boolean (0= no involvement, 1 = involved) |
| **GO:0001947** | Indicates gene’s involvement in heart looping |  | Boolean (0= no involvement, 1 = involved) |
| **GO:0003151** | Indicates gene’s involvement in outflow tract morphogenesis |  | Boolean (0= no involvement, 1 = involved) |
| **GO:0060413** | Indicates gene’s involvement in atrial septum morphogenesis |  | Boolean (0= no involvement, 1 = involved) |
| **GO:0055010** | Indicates gene’s involvement in ventricular cardiac muscle tissue morphogenesis |  | Boolean (0= no involvement, 1 = involved) |
| **GO:0055007** | Indicates gene’s involvement in cardiac muscle cell differentiation |  | Boolean (0= no involvement, 1 = involved) |
| **GO:0060412** | Indicates gene’s involvement in ventricular septum morphogenesis |  | Boolean (0= no involvement, 1 = involved) |
| **GO:0035050** | Indicates gene’s involvement in embryonic heart tube development |  | Boolean (0= no involvement, 1 = involved) |
| **GO:0003148** | Indicates gene’s involvement in outflow tract septum morphogenesis |  | Boolean (0= no involvement, 1 = involved) |
| **GO:0055009** | Indicates gene’s involvement in atrial cardiac muscle tissue morphogenesis |  | Boolean (0= no involvement, 1 = involved) |
| **GO:0003197** | Indicates gene’s involvement in endocardial cushion development |  | Boolean (0= no involvement, 1 = involved) |
| **GO:0003215** | Indicates gene’s involvement in cardiac right ventricle morphogenesis |  | Boolean (0= no involvement, 1 = involved) |
| **GO:0003203** | Indicates gene’s involvement in endocardial cushion morphogenesis |  | Boolean (0= no involvement, 1 = involved) |
| **GO:0048738** | Indicates gene’s involvement in cardiac muscle tissue development |  | Boolean (0= no involvement, 1 = involved) |
| **GO:0003181** | Indicates gene’s involvement in atrioventricular valve development |  | Boolean (0= no involvement, 1 = involved) |
| **GO:0060347** | Indicates gene’s involvement in heart trabecula formation |  | Boolean (0= no involvement, 1 = involved) |
| **GO:0072358** | Indicates gene’s involvement in cardiovascular system development |  | Boolean (0= no involvement, 1 = involved) |
| **GO:0055008** | Indicates gene’s involvement in cardiac muscle tissue morphogenesis |  | Boolean (0= no involvement, 1 = involved) |
| **GO:0003211** | Indicates gene’s involvement in cardiac ventricle formation |  | Boolean (0= no involvement, 1 = involved) |
| **GO:0002376** | Indicates gene’s involvement in immune system process |  | Boolean (0= no involvement, 1 = involved) |
| **GO:0007268** | Indicates gene’s involvement in chemical synaptic transmission |  | Boolean (0= no involvement, 1 = involved) |
| **GO:0006954** | Indicates gene’s involvement in inflammatory response |  | Boolean (0= no involvement, 1 = involved) |
| **GO:0007411** | Indicates gene’s involvement in axon guidance |  | Boolean (0= no involvement, 1 = involved) |
| **GO:0007605** | Indicates gene’s involvement in sensory perception of sound |  | Boolean (0= no involvement, 1 = involved) |
| **GO:0050885** | Indicates gene’s involvement in neuromuscular process controlling balance |  | Boolean (0= no involvement, 1 = involved) |
| **GO:0006955** | Indicates gene’s involvement in immune response |  | Boolean (0= no involvement, 1 = involved) |
| **GO:0007283** | Indicates gene’s involvement in spermatogenesis |  | Boolean (0= no involvement, 1 = involved) |
| **GO:0002250** | Indicates gene’s involvement in adaptive immune response |  | Boolean (0= no involvement, 1 = involved) |
| **GO:0030317** | Indicates gene’s involvement in flagellated sperm motility |  | Boolean (0= no involvement, 1 = involved) |
