# Supplementary figures and images for: A machine learning classifier to identify and prioritise genes associated with murine cardiac development
Source: PLoS Genet. 2026 Feb 10;22(2):e1011489. doi: 10.1371/journal.pgen.1011489 (PMC12919933; doi:10.1371/journal.pgen.1011489)

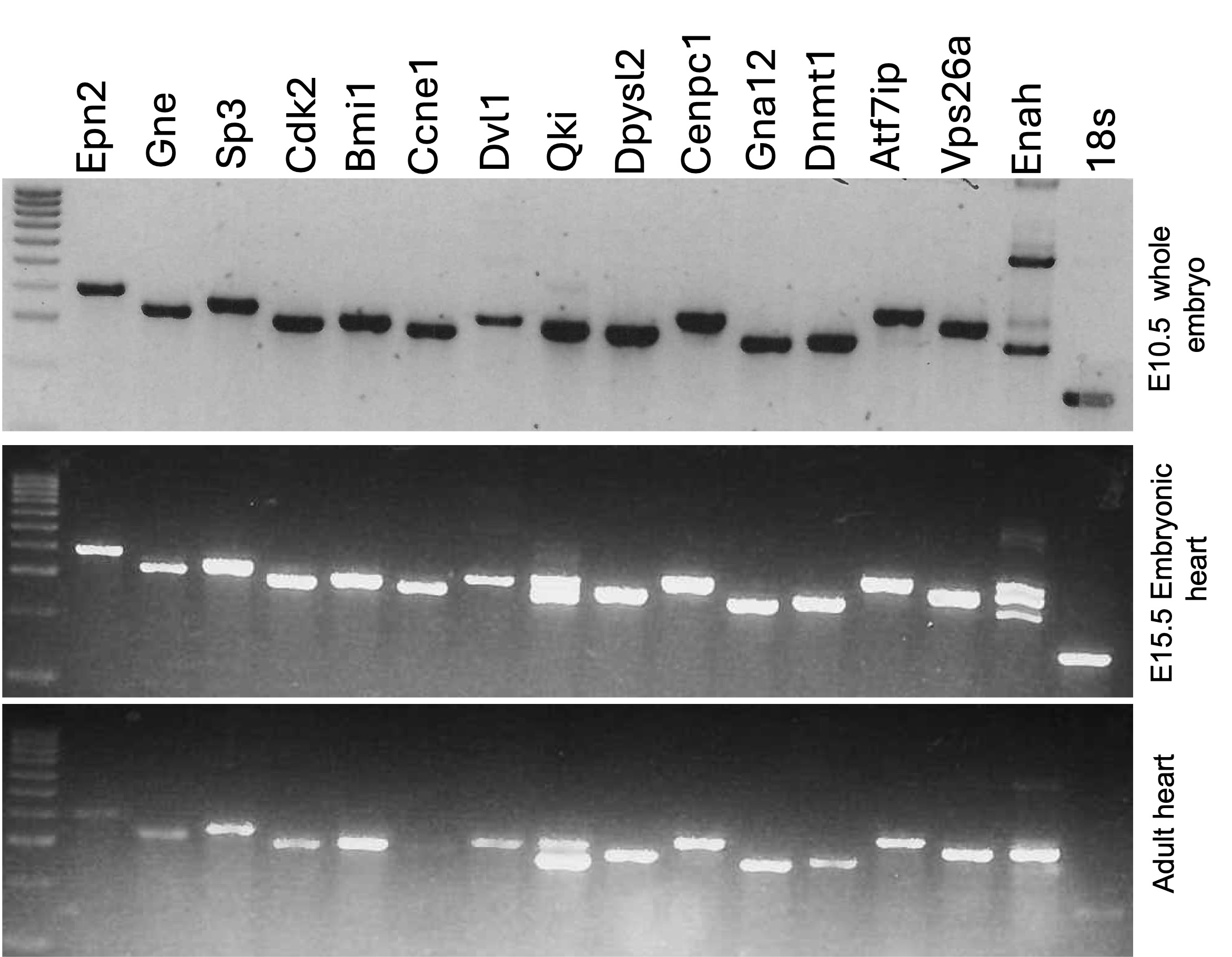

Supplement: S1 Fig — Analysis was performed on cDNA isolated at different stages, including (A) E10.5 mouse whole embryo, (B) E15.5 mouse embryonic heart, and (C) adult mouse heart samples. Genes examined include Epn2, Gne, Sp3, Cdk2, Bmi1, Ccne1, Dvl1, Qki, Dpysl2, Cenpc1, Gna12, Dnmt1, Atf7ip, Vps26a, and Enah. The expression of 18S cDNA was used as a positive control for the presence of a cDNA template. DNA ladder 100 bp (Bioline). (TIFF) [file pgen.1011489.s019.tiff]

E16.5 hearts

*Atf7ip*<sup>f/f</sup>

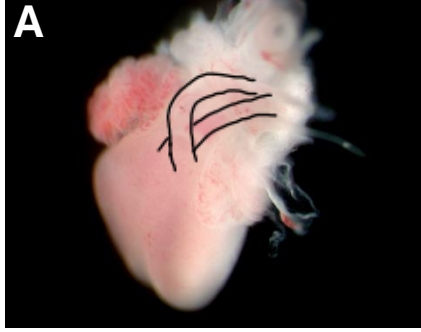

*MHC-Cre; Atf7ip*<sup>f/f</sup>

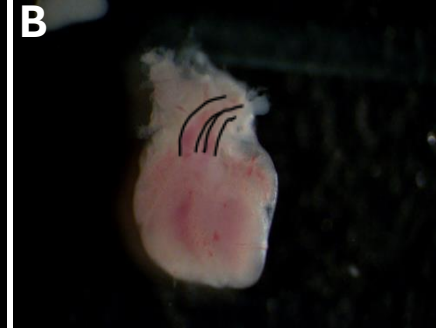

*MHC Cre; Atf7ip*<sup>f/f</sup>

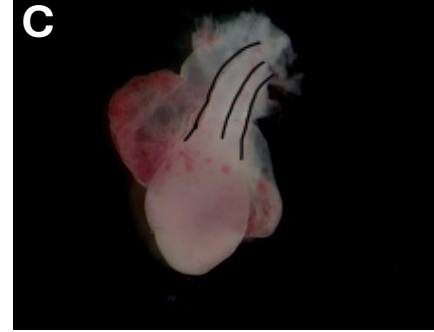

H&E staining

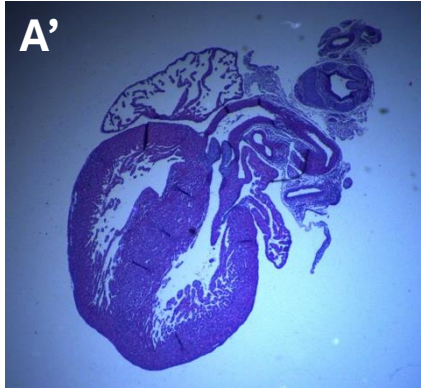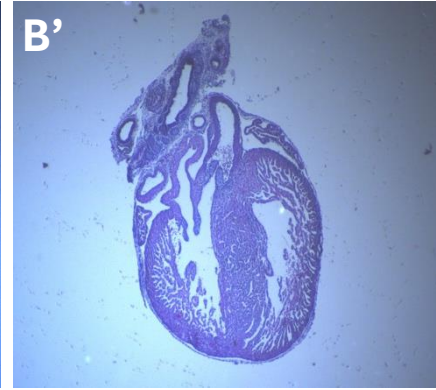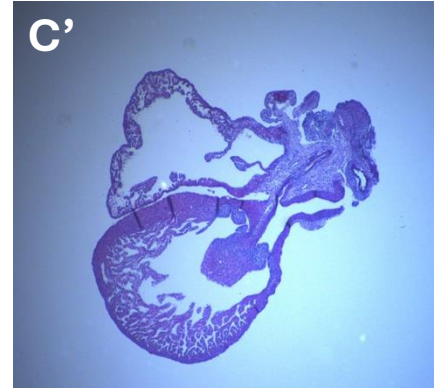

Supplement: S2 Fig — Representative images of E16.5 embryonic hearts and corresponding H&E-stained heart sections. (A–C) Whole-heart images from control and Atf7ip myocardial-specific knockout embryos. (A′–C′) H&E-stained sections of the same hearts. Images show cardiac structural abnormalities, including double outlet right ventricle (DORV) and single ventricular morphology (magnification x2.5). (PDF) [file pgen.1011489.s020.pdf]
